# Combined Multireference–Multiscale Approach to the Description of Photosynthetic Reaction Centers

**DOI:** 10.1021/acs.jctc.4c00578

**Published:** 2024-08-08

**Authors:** Maria Drosou, Sinjini Bhattacharjee, Dimitrios A. Pantazis

**Affiliations:** Max-Planck-Institut für Kohlenforschung, Kaiser-Wilhelm-Platz 1, 45470 Mülheim an der Ruhr, Germany

## Abstract

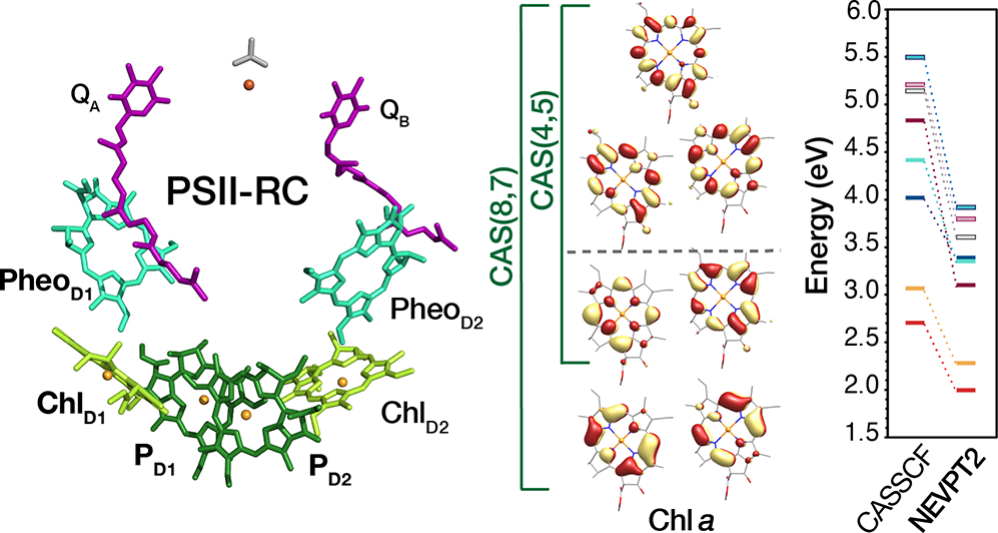

A first-principles description of the primary photochemical processes that drive photosynthesis and sustain life on our planet remains one of the grand challenges of modern science. Recent research established that explicit incorporation of protein electrostatics in excited-state calculations of photosynthetic pigments, achieved for example with quantum–mechanics/molecular–mechanics (QM/MM) approaches, is essential for a meaningful description of the properties and function of pigment–protein complexes. Although time-dependent density functional theory has been used productively so far in QM/MM approaches for the study of such systems, this methodology has limitations. Here we pursue for the first time a QM/MM description of the reaction center in the principal enzyme of oxygenic photosynthesis, Photosystem II, using multireference wave function theory for the high-level QM region. We identify best practices and establish guidelines regarding the rational choice of active space and appropriate state-averaging for the efficient and reliable use of complete active space self-consistent field (CASSCF) and the N-electron valence state perturbation theory (NEVPT2) in the prediction of low-lying excited states of chlorophyll and pheophytin pigments. Given that the Gouterman orbitals are inadequate as a minimal active space, we define specific minimal and extended active spaces for the NEVPT2 description of electronic states that fall within the Q and B bands. Subsequently, we apply our multireference–QM/MM protocol to the description of all pigments in the reaction center of Photosystem II. The calculations reproduce the electrochromic shifts induced by the protein matrix and the ordering of site energies consistent with the identity of the primary donor (Chl_D1_) and the experimentally known asymmetric and directional electron transfer. The optimized protocol sets the stage for future multireference treatments of multiple pigments, and hence for multireference studies of charge separation, while it is transferable to the study of any photoactive embedded tetrapyrrole system.

## 1 Introduction

**Figure 1:**
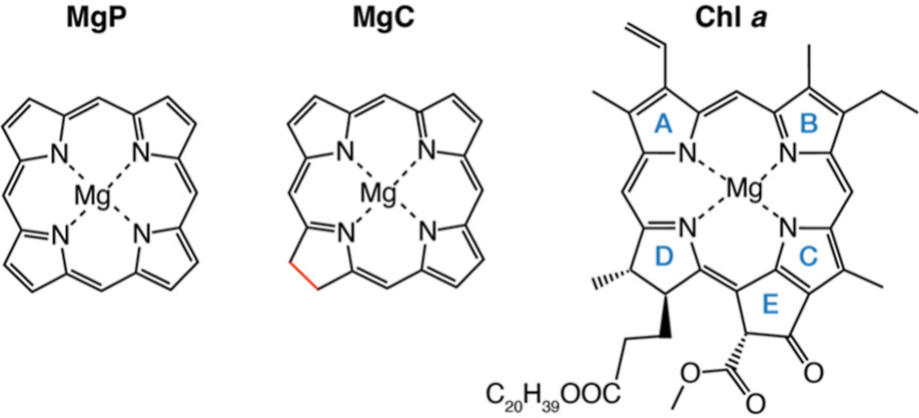
Schematic depiction of magnesium porphyrin (MgP), magnesium chlorin (MgC), and chlorophyll *a* (Chl *a*).

Photosynthesis sustains Earth’s biosphere by using solar energy to drive essential biochemical reactions. The fundamental processes involved in photosynthesis include light harvesting, excitation energy transfer (EET), charge separation, and electron transfer.^1^ The majority of these functions are carried out by a limited variety of pigments embedded in membrane-bound protein complexes. These pigments are of the chlorin and bacteriochlorin type, cyclic tetrapyrrole derivatives of the parent porphyrin system (Figure 1).^2−4^ The electrostatics and conformational dynamics of the protein matrices that host these pigments tune their local properties and enable the emergence of collective phenomena such as directional EET and charge separation, which would be otherwise impossible.

The computational modeling of these systems faces a number of distinct and independent challenges that must be adequately and simultaneously addressed in order to obtain meaningful insights into chemical and physical properties.^5−7^ A major challenge is the treatment of the environment, which is necessary because the protein matrix uniquely diversifies otherwise chemically identical pigments and creates the possibility for cooperative behavior. This challenge can be addressed by multiscale approaches such as those that involve quantum chemical treatment of the pigments coupled with a classical force-field based description of the protein (QM/MM).^5−27^ Another distinct challenge is the description of excited states of individual pigments or pigment assemblies i.e., the choice of QM method within the QM/MM treatment.^14,28−34^ Although time-dependent density functional theory (TD-DFT) has been used productively in this context,^11−13,35−45^ it has acknowledged limitations and weaknesses,^46,47^ including problems with excited states of charge transfer character^48^ and sensitivity of the results on the choice of density functional. This typically necessitates the selection of a DFT method in a case-specific manner, based on benchmarking against higher-level methods.^37,49,50^

This situation has motivated the exploration of more robust wave function methods for (bacterio)chlorin pigments. Among those are the symmetry-adapted cluster configuration interaction (SAC–CI),^51,52^ DFT/multireference configuration interaction,^53^ as well as various coupled cluster-based approximations,^50,54−56^ among which the domain-based local pair natural orbital similarity transformed equation of motion coupled cluster (DLPNO–STEOM-CCSD) method has been used in a multiscale QM/MM framework to provide reference-quality electrochromic shifts^56^ for lower-level coupled-cluster approaches and TD-DFT. On the other hand, multireference methods can, in principle, provide the most accurate description of excited states of strongly correlated π conjugated systems, which often have multiple near-degenerate electronic states with genuine multireference character.^57,58^ Besides, multireference methods are the only way to obtain an explicit description of the wave function of each individual state, which provides the highest level of insight into electronic structure. Contrary to single-reference ab initio approaches, these methods can predict and describe double excitations^59,60^ and can provide detailed and direct insight into the nature of excited states at a wave function level. In addition, their potentially lower cost relative to highly accurate single-reference wave function-based approaches enables their application on larger multichromophoric systems and even in ab initio dynamics approaches.^61^ Although multireference approaches in various forms and implementations have long being pursued,^31,33,52,53,61−66^ deploying these methods in large-scale applications remains difficult due to their inherent complexity and the sensitivity of numerical results upon a multitude of methodological choices.

**Figure 2:**
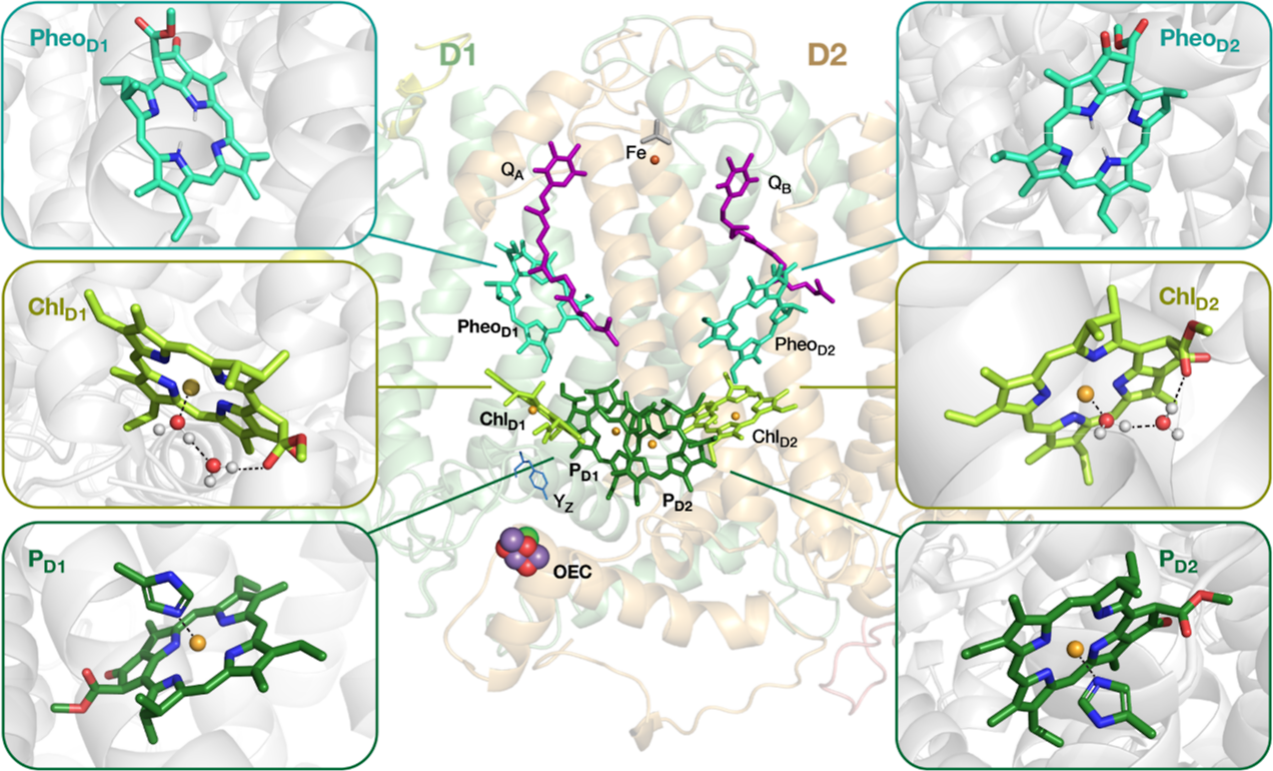
Depiction of the arrangement of pigments in the RC of a PSII monomer. Protein D1 is shown in green and D2 in brown. The structures of the six chromophores in their protein environment are shown in the insets. The structures as shown correspond to the definition of the QM regions used in our QM/MM calculations. The molecular structure of Pheo *a* lacks the central Mg ion and instead has protons on two nitrogens.

Here we develop and establish a multireference–multiscale protocol for the description of low-lying excited states of protein-embedded chlorin-based pigments relevant to photosynthetic reaction centers (RCs) and apply it to the RC in Photosystem II (PSII) (Figure 2). The PSII-RC is an assembly of four protein-embedded Chlorophyll *a* (Chl_D1_, Chl_D2_, P_D1_, P_D2_) and two Pheophytin *a* (Pheo_D1_, Pheo_D2_) pigments,^67^ responsible for the primary charge separation that drives water oxidation,^4,68,69^ the process that sustains all aerobic life forms. Multireference methods have been previously employed for the study of electronic spectra of highly symmetric parent compounds of (magnesium) chlorin, i.e. (magnesium) porphyrin (Figure 1),^52,70−74^ and comparatively less on chlorophylls^61,63,75,76^ and bacteriochlorophylls.^33,65,77,78^ Technical choices such as the size and composition of the active space (AS) and the number of states included in the state-averaged orbital optimization significantly affect the predicted energies and nature of the calculated states. Such studies have also stressed the critical importance of including dynamic correlation, typically via perturbative approaches, but often empirical parameters have to be employed to account for inherent shortcomings of the latter.^79,80^ In this work, we address these fundamental challenges, following a systematic and chemically oriented approach for the rational determination of all parameters involved.

Our primary aim is to establish a solid, transparent, and transferable methodological framework using complete AS self-consistent field (CASSCF) and the *N*-electron valence state perturbation theory (NEVPT2)^81−85^ in a multilevel QM/MM framework. Recent studies by our group demonstrated that protein matrix electrostatics are almost exclusively responsible for the differentiated excited state properties of PSII-RC pigments (and chiefly responsible for the same effect in intrinsic antenna complexes),^6,11−13,41^ and thus for the ability to convert excitation energy to charge separation, and for the directionality of primary and secondary electron transfer processes. We utilize multiscale models of the PSII monomer and revisit this problem from the point of view of multireference theory. This work presents the first application of a rationally optimized multireference CASSCF/NEVPT2 protocol to a photosynthetic RC in a multiscale QM/MM framework. The second aim of this work is to compare and contrast the physical insights into the properties and function of the PSII-RC obtained by single- and multireference approaches. Finally, we aim to ensure extensibility of the approach in order to address in the future other major open questions^6,14,86,87^ on the fundamental processes that determine the function of the RC, such as the nature of higher energy excitations and of multichromophoric charge transfer states that define the primary charge separation events.

## 2 Methodology

### 2.1 Model Setup

The multiscale model used in this work is based on the high-resolution crystal structure of PSII from *Thermosynechococcus vulcanus* (PDB ID: 3WU2).^67^ The setup consists of the complete PSII monomer embedded within a POPC lipid bilayer of dimension 176 × 176 Å^2^ using Packmol-Memgen^88^ and solvated with a TIP3P water box.^89^ The system is neutralized with appropriate number of counterions and a 0.15 M salt buffer. For the standard amino acid residues, waters and lipid bilayer we used the standard AMBERff14SB,^90^ TIP3P and LIPID17 force fields,^91^ respectively. The partial charges and force field parameters for organic cofactors (plastoquinones, carotenoids, structural lipids) were obtained using GAFF2.^92^ Parameters for the oxygen-evolving complex were taken from prior work in our group.^11^ Parameters for chlorophylls, nonheme Fe and for heme-b were obtained from the literature,^93−95^ while those for heme-c were derived in this work. The nonbonded parameters for the metal ions were based on their respective oxidation states using data sets available for the TIP3P water model.^96^ For Na^+^ and Cl^–^ ions, we used the Joung–Cheatham parameters compatible with the TIP3P water model.^97,98^ A stepwise minimization protocol was employed to remove energetically unfavorable geometric clashes in the system. In the equilibration phase, heating from 10 to 100 K in a succession of 5 ps in the *NVT* ensemble was followed by further heating from 100 to 303 K in the *NPT* ensemble for a total of 125 ps. The temperature during this step is maintained using Langevin dynamics^99^ with a collision frequency of 5 ps^–1^ and the pressure was maintained at 1 bar using the Berendsen barostat.^100^ During equilibration, the Cα atoms of amino acids were restrained with a harmonic force constant of 20 kcal mol^–1^ Å^–2^. We employed the SHAKE algorithm^101^ to constrain the bonds involving hydrogens, therefore a time step of 2 fs could be used. The electrostatic interactions were treated using the Particle Mesh Ewald approach^102^ with a 10 Å cutoff. The AMBER20 package^103^ was used to perform energy minimizations and equilibration dynamics.

For further QM/MM excited state calculations we chose two distinct MD snapshots: one before the heating dynamics, which resembles a “crystal-like” protein configuration, and the other immediately after the initial equilibration. The choice of these snapshots is dictated by the fact that we aim to explicitly investigate and account for the protein conformational changes as well as for distinct ring distortions on the excited state properties of the pigments. Our methodology therefore enables us to characterize how the changes in the internal geometry and surrounding protein configuration changes may explicitly tune the excited state properties of RC pigments.

### 2.2 Geometry Optimizations

In the current study we used three different types of model (Table 1) depending on the QM/MM optimization protocol and the respective protein configuration obtained from the previous step. Model type **A** represents the absolutely minimal departure from the crystallographic model, in which the MM region has not been relaxed except for hydrogens, lipids, and the water box. At the same time, the heavy atoms in the QM region are fixed in their crystallographic coordinates (of course, the hydrogen atoms which are absent or undefined in crystallographic structures are always optimized). Model type **B** retains the “crystal-like” configuration of the protein but in this case the QM region is fully optimized. Finally, in model type **C** the protein configuration is taken after performing the initial MD equilibration and the QM region is again fully optimized within this equilibrated protein matrix.

**Table 1:** Different Types of Models Discussed in the Present Work with Respect to the Conformation and Geometry Optimization of the QM and MM Regions^a^

model	QM region	MM region
**A** (XRD|XRD)	Only H atoms optimized; heavy atoms fixed at crystallographic positions.	Only hydrogens, lipids and water box minimized; “crystal-like” configuration.
**B** (QM|XRD)	All QM atoms optimized.	Only hydrogens, lipids and water box minimized; “crystal-like” configuration.
**C** (QM|MD)	All QM atoms optimized.	After initial MD equilibration.

a XRD implies use of crystallographic coordinates.

For all QM/MM calculations the complete PSII monomer and all waters around the protein were retained (7 Å bulk-region, in total 8000 water molecules including internal cavity waters) and an appropriate number of Na^+^ ions were included to maintain overall neutrality. The final atom count for each QM/MM setup was 76,035.

All QM/MM calculations were performed using the electrostatic embedding scheme in ORCA 5.^104^ The hydrogen link atom approach was employed to cut through C–C covalent bonds and the charge-shift method was used to avoid overpolarization of the QM region. Along with the chlorin macrocycles, the axially coordinated ligands to the Mg^2+^ were treated at the QM level. The phytyl chains were included up to C-17 (truncated as a methyl group) and the rest of the chain was kept in the MM region. The complete system was further subdivided into two parts: an active region consisting of atoms within both the QM and MM regions that are free to move during the optimization, while the remaining MM atoms are static, contributing to the electrostatics. This structurally active composite region comprised complete amino acid residues and waters within 10 Å from the center of each chlorin ring.

The Perdew–Burke–Ernzerhof (PBE) functional^105^ and its hybrid version PBE0^106^ were used to optimize the QM regions using the def2-TZVP basis set,^107^ along with D3(BJ) dispersion corrections.^108^ Geometry optimizations of isolated chromophores in vacuo were also performed to enable specific comparisons. The resolution of identity along with the “chain of spheres” approximation for exchange (RIJCOSX)^109^ were used in all calculations. The def2/J auxiliary basis sets^110^ were used for all atoms. Tight SCF convergence criteria and dense DFT integration grids (DefGrid2 in ORCA convention) were used throughout.

### 2.3 TD-DFT Calculations

Vertical excitation energies (6 roots) were computed on the 3 models of QM/MM geometries for each RC pigment using full TD-DFT i.e., without the Tamm–Dancoff approximation. We used the range-separated ωB97X-D3(BJ) functional^111^ (modified version of ωB97X-V^112^ with D3(BJ) dispersion correction) along with the def2-TZVP basis sets. The RIJCOSX approximation and the corresponding auxiliary basis sets were used throughout. VeryTightSCF convergence criteria were applied, along with dense integration grids (DefGrid2). To calculate the protein electrochromic shifts, the site energies for each RC pigment were additionally computed in vacuo, i.e., in the absence of MM point charges, while retaining the QM/MM-optimized geometry for the QM region.

### 2.4 Multireference Calculations

Single-point CASSCF calculations with the def2-TZVP basis sets were performed on the optimized geometries. All CASSCF calculations in this work were state-averaged (SA), which means that the orbitals were optimized for multiple states (roots) simultaneously, i.e. the ground state and a given number of excited states, ranging from 1 to 9. State averaging is essential for achieving the highest possible accuracy in energy differences, which is the essence of the problem in the present case, similar to other types of problem where multireference approaches have been used.^113−116^ For a given number of roots, we first performed the SA-CASSCF(4,4) calculation i.e. four electrons in four orbitals, starting from converged ground-state Kohn–Sham (PBE0) orbitals. Subsequently, starting from the CASSCF(4,4) orbitals, the AS was gradually expanded in steps of one, two, or three occupied or unoccupied orbitals, and the effect of including a specific orbital was studied in detail. In this way we examine the stability of the multireference description in terms of excitation energies, oscillator strengths, and character of the excited states with respect to the size and nature of the AS, as well as with respect to the number of roots over which state-averaging is performed.

In all calculations the effects of dynamic correlation were included with multireference perturbation theory using the NEVPT2 method in the strongly contracted (SC) variant.^81−83^ Since CASSCF energies are significantly (>0.5 eV) higher than experimental values irrespective of the chosen AS or number of averaged states, only NEVPT2 energies, oscillator strengths, and transition dipole moments are presented in the text, unless stated otherwise. NEVPT2 calculations were performed using the def2-TZVP basis sets. We note that test calculations using quadruple-ζ def2-QZVP and def2-QZVPP basis sets showed that the def2-TZVP results are practically converged with respect to basis set size. In contrast to another popular multireference perturbative dynamic correlation approach, CASPT2,^117−120^ which typically needs ad hoc and nontransferable empirical correction factors to compensate for inherent inadequacies,^79,121^ NEVPT2 has considerable fundamental advantages owing to the fact that it is based on the Dyall Hamiltonian,^122^ and is adopted here from the outset as the optimal choice for a generally applicable protocol. Notably, the results obtained from SC-NEVPT2 and fully internally contracted (FIC) NEVPT2^81^ were very similar (Table S9).

## 3 Results and Discussion

### 3.1 Definition of the Multireference Approach

The first goal of this work is to explore in detail the methodological parameters involved in describing the target pigments using a CASSCF/NEVPT2 protocol, targeting at first the description of the nature, energies, and oscillator strengths of primarily the two lowest energy electronic transitions (S_1_ and S_2_) that form the Q-band, while ensuring the rational extensibility of the protocol to higher states. To this end, a priority is to determine the minimal AS and number of states (roots) over which the CASSCF orbital optimization should be averaged to ensure a proper description of the ground state as well as S_1_ and S_2_ states in the subsequent NEVPT2 treatment that accounts for dynamic correlation. To achieve this, we systematically explore different AS compositions and examine the stability of the results with respect to the number of averaged states.

The absorption spectra of porphyrin derivatives^123,124^ in the low-energy region comprise the Q-band, which includes the Q_*y*_ and Q_*x*_ transitions, and the Soret (B) band. Herein, we develop our CASSCF/NEVPT2 protocol on Chl *a*, for which the gas phase absorption spectrum is available,^125^ and we therefore avoid additional complications related to the treatment of solvation effects. The Q-band of Chl *a* comprises two transitions, with vertical excitation energies at 1.99 and 2.30 eV, respectively (values corrected with respect to the band maxima).^56^ The experimental values will be used as a point of reference, but we stress that it is not our primary objective to quantitatively reproduce the experimental gas-phase excitation energies but rather to define methodological requirements for a balanced multireference description.

#### 3.1.1 Rational Selection of the Minimal AS

**Figure 3:**
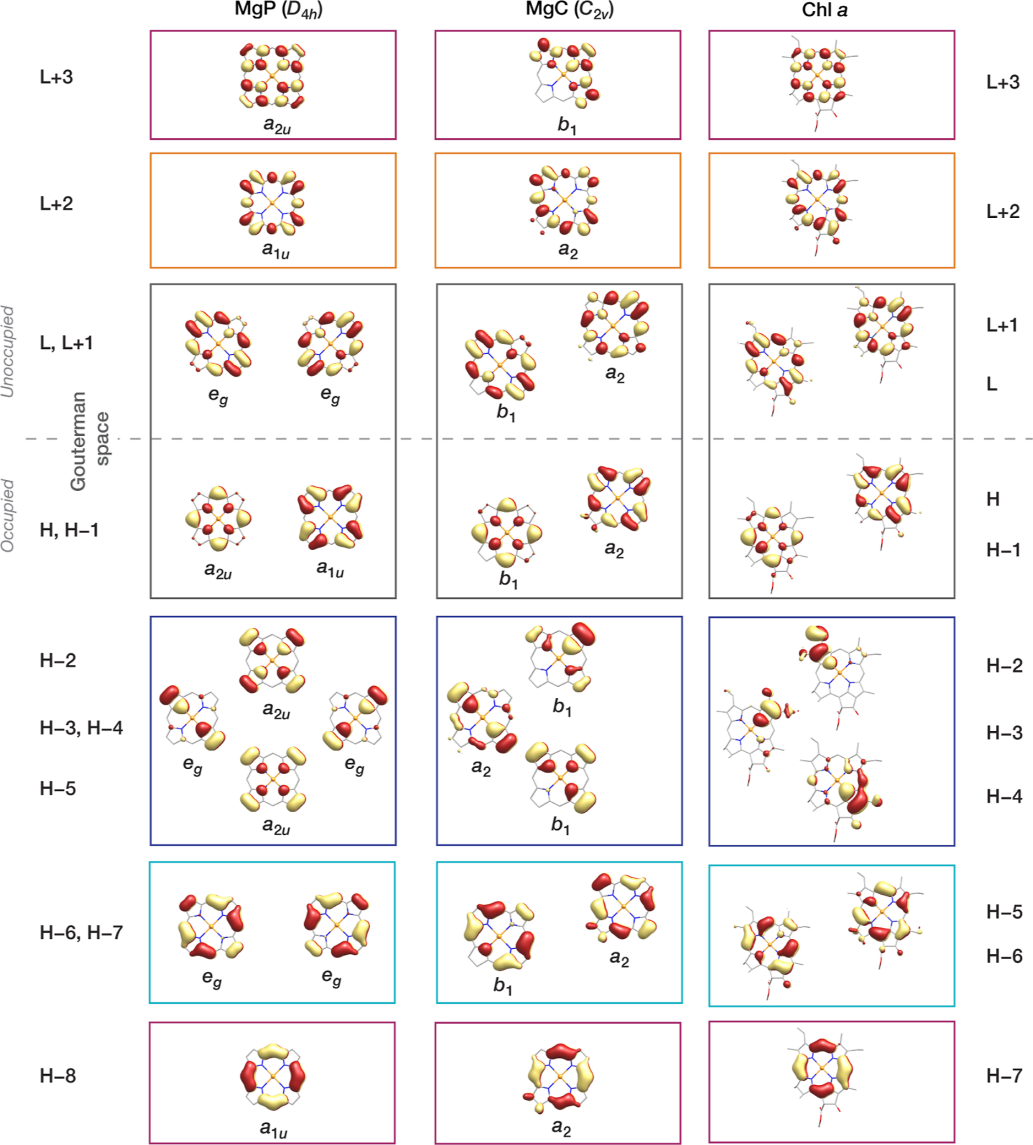
Comparison of magnesium porphyrin (MgP), magnesium chlorin (MgC) and Chlorophyll *a* (Chl *a*) orbitals.

**Figure 4:**
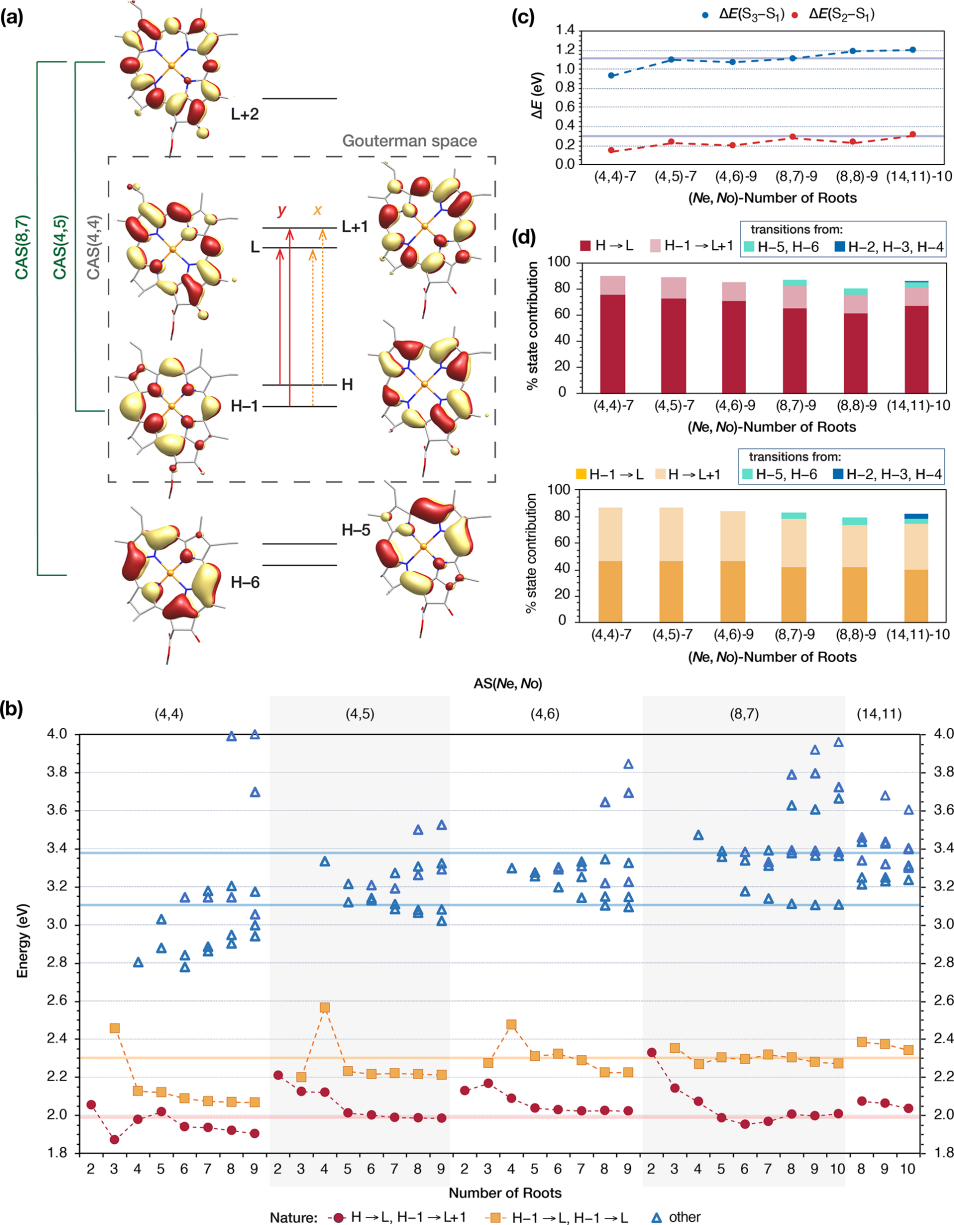
(a) AS orbitals derived from a SA-CASSCF(8,7) calculation state-averaged over 9 roots. (b) NEVPT2 excitation energies for different AS choices and number roots included in the state-averaged orbital optimization. Experimental values derived from the gas-phase absorption spectra of Chl *a* are shown in colored horizontal lines. (c) Calculated energy differences between the first and second (S_2_–S_1_, red) and between the first and third (S_3_–S_1_, blue) excited states using different AS and number of roots. (d) Percentage of excited state contribution for selected CASSCF calculations for S_1_ (red, up) and S_2_ (orange, down) excited states using different AS and number of roots.

According to the Gouterman model for the absorption spectra of porphyrin-like macrocyclic compounds,^124^ the low-energy (1.9–3.5 eV) transitions of porphyrin derivatives are predominantly attributed to transitions between the four frontier orbitals, i.e. highest occupied molecular orbital (HOMO), HOMO – 1, lowest unoccupied molecular orbital (LUMO), and LUMO + 1, which from now on will be denoted H, H – 1, L, and L + 1, respectively. These are π-type orbitals and correspond to various π/π* combinations within the macrocycle, with progressively increasing antibonding character. The H and H – 1 originate from the accidentally degenerate *a*_2*u*_ and *a*_1*u*_ orbitals of the parent porphyrin (*D*_4*h*_ point group), while the L and L + 1 originate from the degenerate *e*_*g*_ orbitals of porphyrin (see Figure 3). In the case of the unsubstituted chlorin ring (*C*_2*v*_ point group) the degeneracies are lifted, with H and L + 1 transforming as *a*_2_ and H – 1 and L transforming as *b*_1_. Excitations between same-symmetry orbitals (H → L + 1 and H – 1 → L) are *x*-polarized in terms of their transition dipole orientation, whereas excitations between different-symmetry orbitals (H → L and H – 1 → L + 1) are *y*-polarized. Even though in the fully substituted Chl *a* itself there are no symmetry elements present anymore, the parentage of the orbitals remains immediately obvious (Figure 3). Therefore, it still makes sense to talk about “Gouterman orbitals” even in the case of Chl *a*, and these indeed can be considered as a chemically motivated choice of a minimal AS for CASSCF calculations. Following the Gouterman model, the minimal AS would thus consist of 4 electrons in 4 orbitals, i.e. (4,4), as shown in Figure 4a (dashed line box). For this AS there are 19 possible singlet configurations and 20 configuration state functions.

Starting from a SA-CASSCF(4,4) orbital optimization over 2 roots, denoted (4,4)-2, i.e. the ground state and the first excited state, we examine the effects of stepwise increasing the number of state-averaged roots up to the point where the NEVPT2 results for S_1_ and S_2_ are stable. The NEVPT2 energies of the excited states calculated using different ASs and different numbers of CASSCF roots are plotted in Figure 4b. The colored symbols correspond to transitions with the same character i.e., red circles are for transitions with predominantly H → L and secondarily H – 1 → L + 1 character, while orange rectangles correspond to transitions with predominantly H – 1 → L and secondarily of H → L + 1 character. The nature of these transitions is consistent with the Q_*y*_ and Q_*x*_ transitions, respectively, as described by the Gouterman model. Higher energy transitions, which are shown in blue triangles, correspond to the Soret region of the spectrum (B-band) and will be discussed in more detail in Section 3.1.3. The colored horizontal lines correspond to the vertical excitation energies from the gas-phase absorption spectra of Chl *a*,^125^ and are shown for orientation. Detailed results, including the energies, oscillator strengths, transition dipole moments, and orbital compositions calculated for different ASs and number of roots are given in Tables S1–S8.

Analysis of Figure 4b starts with the first point on the left, which corresponds to the first excited state derived from the (4,4)-2 calculation. The ground state is dominated (>92%) by the closed-shell configuration, while the first excited state is described as 81% H → L and 12% H – 1 → L + 1. At the CASSCF level, the excitation energy is computed at 2.991 eV (Table S1), which is 1 eV higher than the Q_*y*_ band observed experimentally. NEVPT2 corrects the energy of the CASSCF roots by introducing dynamic σ–π polarization effects,^72^ and brings the first excited state to 2.053 eV, very close to the experimentally observed Q_*y*_ energy (Figure 4b). Large differences between the excitation energies derived from CASSCF and NEVPT2 are observed in all calculations carried out in this work (see Tables S1 and S2 for comparison of CASSCF and NEVPT2 energies for the (4,4) AS). This shows that accounting for dynamic correlation effects is crucial for obtaining meaningful results for porphyrin derivatives,^70,72,75,76^ thus in the remainder of this work we report and discuss only the NEVPT2 energies (complete results from all calculations are provided as Supporting Information). Importantly, in many cases NEVPT2 even changes the order of the CASSCF states, which implies that the number of roots to be included in the initial CASSCF procedure must be treated with utmost care to ensure that the number of states is sufficient to produce stable final (NEVPT2) results.

As shown in Figure 4b, orbital optimization by state-averaging over 3 states to compute both Q-band transitions, i.e. (4,4)-3, predicts the first excited state at 1.870 eV and the second at 2.473 eV, substantially different from the experimental values. In addition, NEVPT2 inverts the first and second CASSCF excited states (Figure S1), which indicates that the first-order CASSCF description is flawed.

We now examine the effect of increasing the number of roots for the (4,4) AS. Including one more state in the orbital optimization, i.e. averaging over 4 roots, gives the S_1_ at 1.976 eV and S_2_ at 2.126 eV. In this case, NEVPT2 maintains the order of the CASSCF states, with S_1_ being predominantly 78% H → L and 14% H – 1 → L + 1, and S_2_ being 48% H – 1 → L and 39% H → L + 1. Moreover, the calculated oscillator strength of the S_1_ (0.437) is significantly larger than that of the S_2_ state (0.001), in agreement with what is expected from experiment. Notably, the third excited state (S_3_) at 2.802 eV is reasonably close (∼0.3 eV) to the first transition of the B-band of the experimental gas-phase absorption spectra, and its oscillator strength (0.939) is larger than that of the S_1_ state, which is also consistent with experiment. Similar to the S_2_ state, S_3_ is also mixed in nature with the leading configuration H – 1 → L (37%) being very close to the second H → L + 1 (29%). The similarity in the nature of S_2_ and S_3_ states explains why both states need to be included in the orbital optimization to achieve a better description of the Q-band.

By increasing the number of roots to 5 and up to 9, the order of the first and second excited states is maintained and the calculated energies and oscillator strengths are relatively stable despite increasing the number of roots (Figure 4b and Table S3). The first excited state is ∼76% H → L and ∼14% H – 1 → L + 1, and the second is ∼47% H → L + 1 and ∼40% H – 1 → L, fully consistent with the Gouterman model. The energy of S_1_ ranges between 1.9 and 2.0 eV, very close to the experimental value. Even though good qualitative description of the second excited state is achieved as well, its energy is ∼2.1 eV, i.e. ∼0.2 eV lower than the experimental estimate.

An alternative way to evaluate excited states description is the energy difference between excited states rather than between an excited state and the ground state. The experimentally determined S_2_–S_1_ and S_3_–S_1_ energy differences are ∼0.3 and ∼1.1 eV, respectively. In Figure 4c, the computed S_2_–S_1_ and S_3_–S_1_ energy differences derived from calculations with different ASs and number of roots, for which the results are relatively stable in terms of energies and compositions, are plotted and compared to experiment (horizontal-colored lines). Figures 4b and c show that CAS(4,4) calculations underestimate the S_2_–S_1_ energy difference. Overall, the minimal (4,4) AS can provide a reasonable qualitative description of the Q-band if at least 4 states are included in the orbital optimization, but it is insufficient to reproduce the energy difference between the states contributing to the Q-band. Interestingly, the (4,4) AS has been considered insufficient for Chl *a*,^76^ but the present results show that it can still provide a reasonable description if SA-CASSCF is performed including more roots. It is not obvious at this point whether these deficiencies of the Gouterman space can be fully compensated when the (4,4) AS is used as the RAS2 subspace in the context of a RASSCF approach.^63^

Since the Gouterman space is insufficient in this sense, we must redefine our concept of a minimal AS. Thus, we examine the effect of expanding the AS by including additional unoccupied or occupied orbitals. The smallest step in this direction is including in the AS one more unoccupied orbital, the L + 2, shown in Figure 3. Notably, this orbital is the one with immediately higher π* character compared to the L and L + 1 (it has 12 nodes, whereas L and L + 1 have 10, and H has 8). In the parent magnesium porphyrin, it is a nondegenerate orbital of *a*_1*u*_ symmetry, while in magnesium chlorin it has *a*_2_ symmetry, the same as the chlorin H orbital (Figure 3).

As shown in Figure 4b, using a (4,5) AS and averaging over 2, 3, or 4 states results in an unstable and energetically inconsistent description of the first two excited states. Even though the compositions of the states are consistent in terms of predominant contributions, the percentages of each contribution vary as much as ∼30% among the (4,5)-2, CAS(4,5)-3, and 4 calculations (Table S4). By contrast, when 5 or more roots are included, the results are stable and conform with experimental observations. SA-CASSCF(4,5) calculations with 7–9 roots give effectively identical descriptions of the first two excited states, with the energy of the first excited state at ∼1.99 eV and composition mainly ∼73% H → L and ∼16% H – 1 → L + 1, and the second excited state at ∼2.22 eV and its composition ∼46% H – 1 → L and ∼40% H → L + 1. Moreover, the excitation energies of the S_3_–S_6_ states are close to those observed for the B_*x*_ and B_*y*_ bands, as shown in Figure 4b. The corresponding S_2_–S_1_ and S_3_–S_1_ energy differences are ∼0.2 and ∼1.1 eV (Figure 4c), and the oscillator strength of S_1_ (0.340) is significantly larger than S_2_ (0.004), in agreement with experiment.

**Figure 5:**
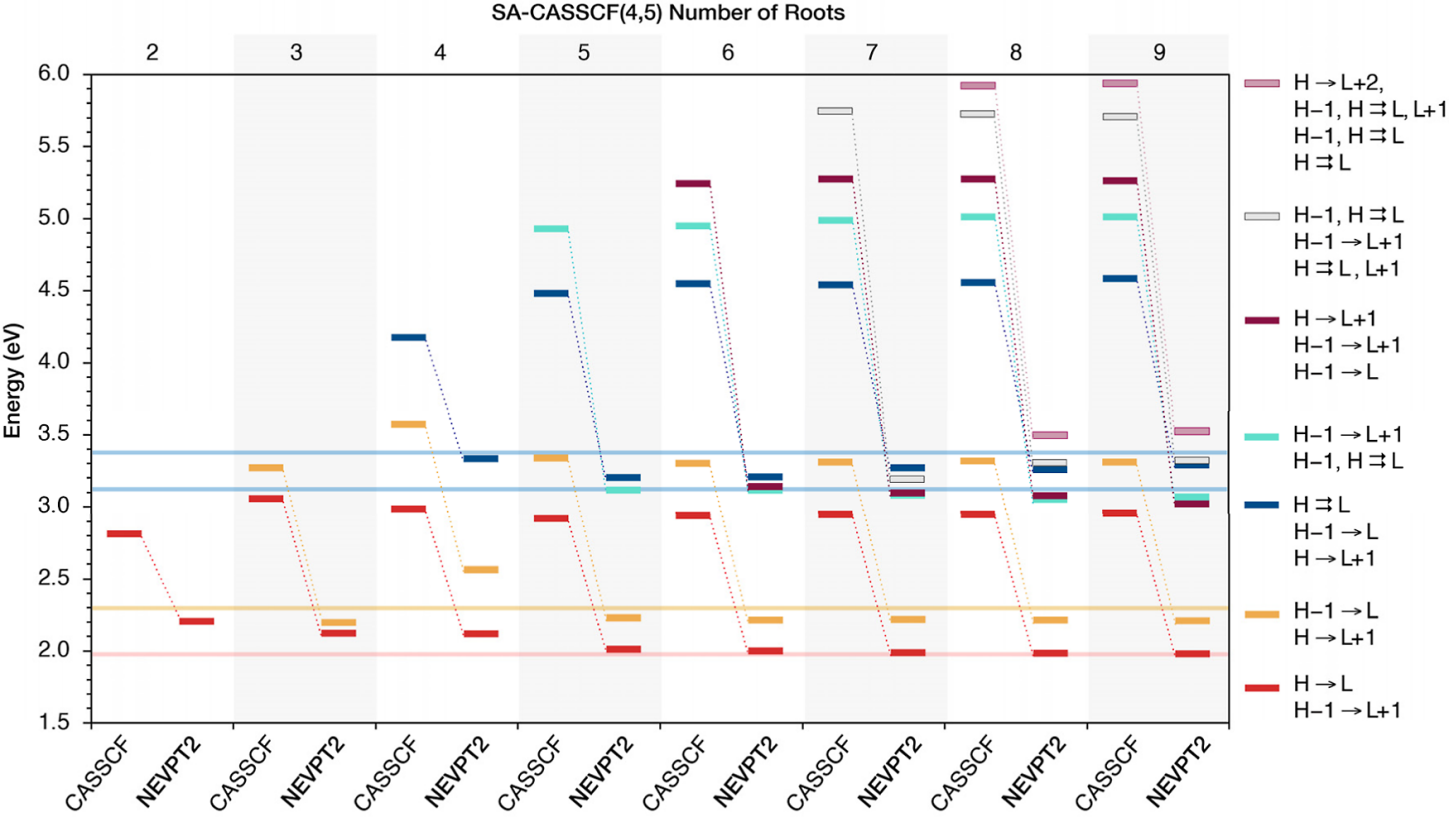
Comparison of NEVPT2 excitation energies of Chl *a* derived from SA-CASSCF(4,5) calculations depending on the number of roots (2–9) included in the state-averaged orbital optimization. The nature of each root in terms of the most important configurations (i.e., with contribution >10% on the root’s wave function) is given on the right. Double excitations are shown with a double arrow symbol. Each color corresponds to the same CASSCF root. The nature of the CASSCF roots is qualitatively the same in terms of the nature of the most important contributions, regardless of the number of roots considered in the state-averaged orbital optimization. NEVPT2 changes the order of higher CASSCF states and the corresponding states are connected with dotted lines and depicted in the same color.

At this point, it is worth examining why the orbitals need to be optimized for at least four excited states (i.e., five roots in total) to get stable and reliable results for the lowest two excited states. Figure 5 shows that in all our SA-CASSCF(4,5) calculations, CASSCF roots 3–6 involve predominantly transitions among the four frontier orbitals, i.e. H → L + 1, H – 1 → L, H → L, and H – 1 → L + 1. Since states S_1_ and S_2_ are composed of transitions of similar nature, a possible explanation is that orbital optimization to accommodate these transitions is needed to get sufficiently good orbitals to describe the first two excited states. Interestingly, including the seventh excited CASSCF state in the state-averaged orbital optimization, i.e. the eighth root in the CASSCF(4,5)-8 calculation, which has a H → L + 2 dominant contribution, does not affect either the CASSCF or the NEVPT2 results (Table S4). Notably, the third CASSCF root in all our CASSCF(4,5) calculations has predominantly a H → L double excitation character (Figure 5). Due to the NEVPT2 reordering, the fourth and fifth CASSCF excited states, which have predominantly single excitation character, become states S_3_ and S_4_ in all CASSCF/NEVPT2 calculations with five or more roots, as they are stabilized by NEVPT2 contrary to the H → L double excitation. Thus, at least five roots need to be included in the orbital optimization to have a state with single excitation character (4th root, H – 1 → L + 1) in the orbital optimization.

Overall, the above analysis leads to a crucial conclusion regarding the fundamental methodological choice for the study of the low energy region of the Chl *a* spectra: a (4,5) AS is the minimal AS required and 7 roots need to be included in a SA-CASSCF to obtain a reliable and stable description of the first two excited states of Chl *a*. Therefore, the choice of the Gouterman AS of four orbitals should be avoided even if only the first and/or second states are of interest.

#### 3.1.2 Expanded ASs

Having determined the minimal requirements for a proper description of the Q-band of Chl *a*, we now examine the effect of expanding the AS. Our main purpose now is to determine how to expand the AS in a balanced way. We begin with expanding the AS in the direction of the unoccupied orbital space. Starting from SA-CASSCF(4,5) orbitals, we add the L + 3 orbital (Figure 3) in the AS, which results in an AS of 4 electrons in 6 orbitals. In the parent magnesium porphyrin, the L + 3 is a nondegenerate orbital of *a*_2*u*_ symmetry, while in magnesium chlorin it is of *b*_1_ symmetry. The NEVPT2 energies of the excited states calculated using the CASSCF(4,6) wave function state-averaged over 2–9 roots are plotted in Figure 4b. Similar to the (4,5) AS, the (4,6) AS with 2–4 roots provides vertical excitation energies for the two lowest excited states that significantly deviate from experiment and are unstable with respect to the number of roots, whereas averaging over more than 5 excited states leads to a more stable and consistent description. The NEVPT2 energies based on the (4,6)-7, (4,6)-8, and (4,6)-9 calculations are very close to those of the (4,5)-7, (4,5)-8, and (4,5)-9 calculations (Tables S4 and S5). The average orbital occupations of the (4,5)-7 and the (4,6)-7 calculations are very similar (Figure S2) and the L + 3 orbital in the (4,6)-7 calculation has an occupation of only 0.02, which reflects the very small contribution to the excited state configurations. Therefore, including the L + 3 orbital in the AS changes neither the qualitative nor the quantitative description of the Q-band of Chl *a* relative to the minimal AS (4,5).

Next, we investigate the effect of expanding the AS in the direction of the occupied orbital space. As shown in Figure 3, the occupied orbitals H – 2, H – 3, and H – 4 of Chl *a* are π orbitals localized on the A, B, and C pyrrole rings, respectively. These correspond to the H – 2, H – 3, and H – 4 orbitals of magnesium chlorin with *b*_1_, *a*_2_, and *b*_1_ symmetry, respectively. In the parent magnesium porphyrin, the H – 2 is a nondegenerate orbital of *a*_2*u*_ symmetry, followed by a pair of degenerate *e*_*g*_ orbitals, and then by a nondegenerate orbital of *a*_2*u*_ symmetry, same as the symmetry of H – 2. Instead of an orbital pair, magnesium chlorin has only the nondegenerate orbital of *a*_2_ symmetry (H – 3), because of the reduced double bond in one of its pyrrole rings.

The smallest step toward increasing the AS in the direction of the occupied orbital space would be including one more occupied orbital. Due to the similar nature and energies of Chl *a* orbitals H – 2, H – 3, and H – 4, we tried several different (6,6) and (6,7) orbital optimizations including only one of these orbitals in the AS. These calculations turned out to be problematic, with the lower occupied orbitals frequently rotating out of the AS during orbital optimizations, and due to unstable results with respect to the number of states included in the state-averaged orbital optimizations. Overall, exploratory calculations with different ASs showed that the AS is not balanced unless H – 2, H – 3, and H – 4 are included simultaneously in AS, i.e. a (10,9) AS (Figure S3).

The NEVPT2 energies, oscillator strengths, dominant contributions, and transition dipole moments of the first and second excited states calculated from selected CASSCF wave functions with different ASs and number roots are given in Table 2. As shown in Table 2, the NEVPT2 energies of the two lowest excited states of the (10,9)-9 wave function are 2.108 and 2.433 eV, deviating more than 0.1 eV from the experimental estimates of 1.99 and 2.30 eV for S_1_ and S_2_, respectively. However, the nature of the Q-band excitations, as well as their oscillator strengths and transition dipole moments, are similar to the minimal (4,5) AS, except for the CI coefficients for the second excited state, which are 46% H – 1 → L and 40% H → L + 1 in the SA-CASSCF(4,5)-7 wave function and 34% H – 1 → L and 36% H → L + 1 in the SA-CASSCF(10,9)-9 wave function. Notably, excited configurations involving excitations from the H – 2, H – 3, and H – 4 orbitals contribute by only ∼1% to the S_1_ and S_2_ states, which suggests that these orbitals are more important for the description of higher excited states than the Q-band.

**Table 2:** NEVPT2 Vertical Excitation Energies (in eV), Oscillator Strengths, Leading Configurations, and Transition Dipole Moments (in Debye) for Chl *a*, Obtained from SA-CASSCF Reference Wavefunctions with Different ASs

		S_1_			S_2_	
	contributions of transitions			contributions of transitions		
AS-roots	*E* (eV)	H → L, H – 1 → L + 1	|μ| (D)	*E* (eV)	H – 1 → L, H → L + 1	|μ| (D)
(4,4)-7	1.933 (0.330)	0.76, 0.15	6.70	2.073 (0.003)	0.47, 0.40	0.65
(4,5)-7	1.988 (0.340)	0.73, 0.16	6.71	2.220 (0.004)	0.46, 0.40	0.72
(4,6)-9	2.021 (0.379)	0.71, 0.15	7.02	2.224 (0.004)	0.47, 0.38	0.72
(10,9)-9	2.108 (0.507)	0.75, 0.10	7.96	2.433 (0.019)	0.34, 0.36	1.45
(8,7)-9	1.995 (0.266)	0.66, 0.16	5.93	2.279 (0.015)	0.42, 0.37	1.31
(8,8)-9	2.019 (0.296)	0.62, 0.15	6.22	2.249 (0.047)	0.42, 0.32	2.36
(14,11)-10	2.035 (0.300)	0.67, 0.14	6.23	2.341 (0.004)	0.40, 0.35	0.71

Next, we examine the possibility of skipping orbitals H – 2, H – 3, and H – 4 and expanding the AS by adding orbitals H – 5 and H – 6 instead (Figure 3), which results in an AS with 8 electrons in 7 orbitals. In the parent magnesium porphyrin these are a pair of degenerate orbitals of *e*_*g*_ symmetry, while in magnesium chlorin they are nondegenerate, one with *a*_2_ and one with *b*_1_ symmetry, respectively. As shown in Figure 4b, the calculated NEVPT2 excited state energies using this (8,7) AS are stable with respect to the number of averaged states if at least 5 states are included in the orbital optimization, as was also observed using the smaller ASs (also Table S6). The results derived when averaging over 8–10 states are practically identical, with less than 0.011 eV variation between the NEVPT2 energies of the S_1_ and S_2_ states of the (8,7)-8, (8,7)-9, and (8,7)-10 calculations. The calculated vertical excitation energies—both for the Q-band and the B-band—are in very close agreement with experimental values (Figure 4b). As shown in Table 1, the CASSCF(4,5)-7 and (8,7)-9 descriptions of the two lowest excited states are very similar in terms of energies, oscillator strengths, as well as state contributions (see also Figure 4d where the dominant state contributions to the first and second excited states for a selected wave function of each AS are plotted). The most important difference between these two ASs is in the energy of the second excited state, which is 2.220 eV with the minimal AS (with 7 roots) and 2.279 eV with the (8,7) AS (with 9 roots). Thus, the expanded AS slightly better reproduces the observed S_2_–S_1_ energy difference (Figure 4c). As a final note, very similar results are obtained if the L + 3 orbital is added in the AS, i.e. with an (8,8) AS (Table 1; optimized orbitals are shown in Figure S4). Similar to the SA-CASSCF(4,6)-7 calculation, the L + 3 orbital has a very small (0.02) average orbital occupation. The results confirm that the (8,7) AS provides an exact description of the Q-band, imply that the multireference protocol can be adapted for targeting higher excited states, and enhance confidence in the robustness of the description derived from the minimal (4,5) AS.

Since H – 2, H – 3, and H – 4 orbitals need to be included simultaneously in the AS, the immediately larger AS than the (8,8) is a (14,11) AS (Figure S5). Figure 4b and Table 2 show that the NEVPT2 vertical excitation energies using the (14,11)-10 calculation are very close to those of the minimal AS, i.e. (4,5)-7 calculation, with the first giving slightly higher energies, by ∼0.05 eV for the first excited state and by ∼0.1 eV for the second excited state. We note that the SA-CASSCF(14,11) orbital optimization was possible if at least 8 excited states were included (Table S7). Interestingly, the state contributions plotted in Figure 4d show that in the (14,11)-10 calculation, transitions involving orbitals H – 5 and H – 6 (light-blue areas) contribute to the first excited state significantly more than transitions involving orbitals H – 2, H – 3, and H – 4 (dark blue area), i.e. 4.0 and 0.7%, respectively. By contrast, the contributions to the second excited state are similar, 3.3 and 3.9%, respectively. Therefore, despite being energetically lower, H – 5 and H – 6 contribute more significantly to the Q-band than H – 2, H – 3, and H – 4. Finally, we note that configurations involving excitations from the H – 7 orbital (Figure 3) do not contribute significantly to any of the computed states (S_1_–S_9_), as shown using (16,12) and (16,13) ASs (Figure S5 and Table S8). Expanding the AS beyond this point is expected to deteriorate the CASSCF description of the Q-band due to the need to average the orbital optimization over an even larger number of roots. Thus, the (14,11) AS includes all occupied and unoccupied orbitals relevant to the description of the S_1_ and S_2_ states, and the similarity of the Q-band description to that obtained from the (8,7) and (4,5) ASs confirms that the Q-band description is stable with respect to the size of the AS.

We conclude this section with some general remarks on our overall results. First, CASSCF calculations using all ASs considered in this study provide qualitatively the same description of the Q-band of Chl *a* in terms of dominant excited configurations, consistent with the Gouterman model, provided that five or more states are included in the state-averaged orbital optimization (Figure 4d). Second, in all of our SA-CASSCF calculations, the ground state is dominated (at least 83%) by the closed-shell configuration, which suggests that the ground state of Chl *a* has a single-reference character. This is in agreement with another SA-CASSCF study,^76^ but contrary to a more recent SA-RASSCF description, which suggests that Chl *a* has significant multireference character in the ground state.^63^ Third, for a quantitatively correct description of the Q-band, a minimal AS of four electrons in five orbitals is needed, and the state-averaged orbital optimization should be performed over seven states. Fourth, the excitation energies are converged with respect to the basis set size (triple-ζ), stable independent of the chosen NEVPT2 variant (Figure S7), and consistent with experimental values. Finally, our results using expanded ASs highlight that AS expansion should be carried out carefully, considering the nature and symmetry of the active and virtual orbitals, and that a balanced AS cannot be built by successively adding pairs of π and π* orbitals or by purely energetic considerations, but it is case-specific and should be guided by chemical reasoning. We note that unlike the stable and consistent description of the Q-band with the different ASs, higher excited states are described differently, both in terms of energies and in terms of the nature of the transitions. Although the aim of this work is to describe the low-energy region of the spectra, it is worth examining what these calculations reveal about the nature of the B-band.

#### 3.1.3 Nature of the Higher Excited States (Soret Band)

**Figure 6:**
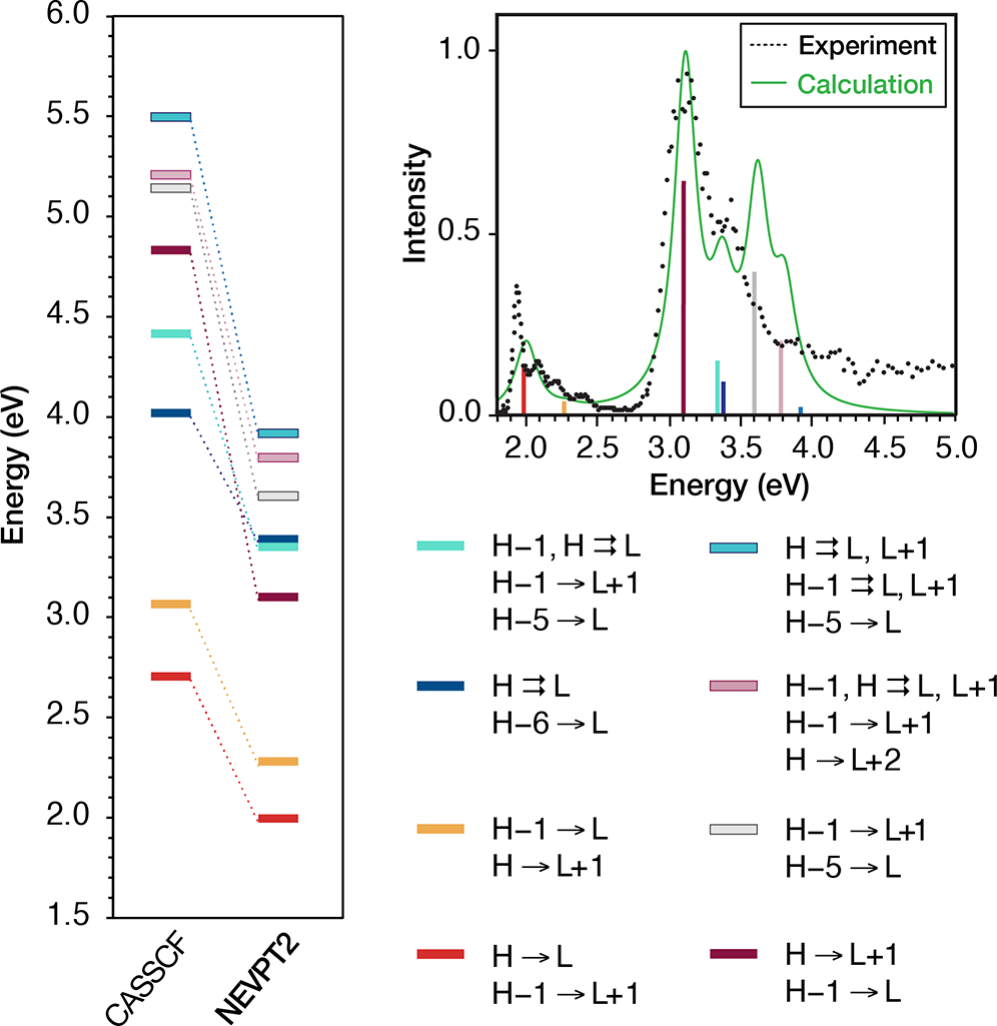
Comparison of CASSCF and NEVPT2 vertical excitation energies for Chl *a* derived from the (8,7)-9 calculation (left) and the stick diagram and simulated spectra (green) using the NEVPT2 energies and oscillator strengths compared with the experimental gas-phase absorption spectra (black dots) of Chl *a*(125) (right). The nature of each root (orbitals are shown in Figure 4a) in terms of the most important configurations (contribution >10%) is shown bellow the spectra.

In addition to providing a stable and realistic description of the Q-band, the (8,7) AS also provides higher excited states with energies and oscillator strengths consistent with the Soret region of the gas-phase spectra of Chl *a* (Figure 4b). The energies of the CASSCF roots and of the NEVPT2 states from the (8,7)-9 calculation are plotted in Figure 6. The simulated absorption spectra (green line) using these NEVPT2 energies and oscillator strengths nicely reproduce the 1.8–3.5 eV region of the gas-phase spectra of Chl *a* (black dots). The third excited NEVPT2 state at 3.102 eV contains predominantly H → L + 1 and H – 1 → L contributions, in line with previous assignments^51,52,56^ of the B_*x*_ band of porphyrin derivatives that conform to the Gouterman model. However, our results suggest that the second band of the Soret region arises from a pair of close-lying states, S_4_ and S_5_, at 3.351 and 3.388 eV, respectively, which have predominantly double excitation character involving the H – 1, H, and L orbitals. Finally, states S_6_ and S_7_ at 3.605 and 3.795 eV, respectively, also have dominant contributions involving the four frontier orbitals. These results suggest that the B-band contains several transitions with single and double excitation character among the four frontier orbitals.

Interestingly, apart from the four frontier orbitals, important contributions (>10%) to the S_4_–S_6_ are observed from configurations involving excitations from the H – 5 and H – 6 orbitals (Figure 3) to the L orbital. In contrast, the contributions of the L + 2 and L + 3 (see also the results of the CASSCF(8,8)-9 calculation in the Supporting Information) to the B-band is very small. Notably, calculations of magnesium porphyrin and free porphyrin excited states also assign the transitions in the Soret region to excitations to the L and L + 1 Gouterman orbitals, rather than to higher non-Gouterman virtual orbitals.^51,52,70−72,74^

A very different picture is obtained when H – 2, H – 3, and H – 4 (Figure 3) orbitals are also included in the AS. Using the (14,11)-10 orbitals (Figure S5), states S_3_, S_4_, S_5_, and S_7_ which are between 3.2 and 3.5 eV, involve predominantly transitions from the H – 2, H – 3, and H – 4 orbitals to L (analysis in Figure S6). Transitions from the ground state to states S_3_, S_4_, and S_5_ are predicted to be very weak (*f* < 0.01), and S_7_ has an oscillator strength of 0.287, which is smaller than that of S_1_. Therefore, these calculations underestimate the intensity of the transitions in the Soret region. Orbitals H – 2, H – 3, and H – 4 are localized on one or two individual pyrrole rings in Chl *a*, therefore transitions from these orbitals to L and L + 1 have significant charge-transfer character and small oscillator strengths, as discussed by Reimers.^51^ In addition, state S_6_ is a double excitation involving H – 1, H, and L, and its calculated oscillator strength is also small (0.222).

Overall, these calculations yield several states in the Soret region, including Gouterman-type transitions, conventionally ascribed to B_*x*_ and B_*y*_, as well as states with double excitation and significant charge transfer character. Yet, this approach does not appear to be converged with respect to the Soret region, which is also evident by the NEVPT2 reordering of higher CASSCF states (Figures 6 and S6). Therefore, the more complicated nature of the higher excited states suggests that a reliable description of the B band needs a more refined approach than the description of the Q-band. This is beyond our current scope and will be pursued in a separate study.

#### 3.1.4 Application to Other Pigments

In the previous sections we established that the minimal AS for the Q-band of Chl *a* contains four electrons in five orbitals (4,5), while the immediately larger balanced AS is (8,7). Moreover, the results are stable if 7 and 9 roots, respectively, are included in the SA orbital optimization. We now examine the application of this protocol to pigments of the RC of PSII. The RC contains four Chl *a* and two Pheo *a* molecules, symmetrically placed along the D1 and D2 polypeptide chains of PSII (Figure 2). The chlorophylls of the P_D1_/P_D2_ pair are axially ligated with histidine residues and the chlorophylls Chl_D1_ and Chl_D2_ are axially ligated with a single water molecule.

In Table 3 we report the results of excited state CASSCF/NEVPT2 calculations on the gas-phase optimized structures of Chl *a* axially substituted on one side with histidine (Chl *a*-His), of Chl *a* axially substituted on one side with water (Chl *a*-H_2_O), and of Pheo *a*. The nature of the S_1_ and S_2_ states, as well as the transition oscillator strengths, are found to be similar for all pigments. Axial substitution of Chl *a* has negligible effect on the energy of the S_1_ state, and slightly red shifts the S_2_ state by less than 0.1 eV. Pheo *a* has the largest S_2_–S_1_ energy difference (∼0.5 eV) among the pigments, as the energy of S_1_ is ∼0.06 eV lower than that of Chl *a* and S_2_ is ∼0.15 eV higher. Regarding the minimal AS, the NEVPT2 vertical excitation energies using the corresponding (4,5)-7 and (8,7)-9 wave functions differ by less than 0.02 eV for the S_1_ state and by less than 0.1 eV for the S_2_ state for all pigments (see also Figure S8). Therefore, the AS requirements of the axially substituted Chl *a* and of Pheo *a* are the same as those of Chl *a*.

**Table 3:** NEVPT2 Vertical Excitation Energies (in eV), Oscillator Strengths, Leading Configurations, and Transition Dipole Moments (in Debye) for Chl *a*, for axially substituted Chl *a* with His and with H_2_O, and for Pheo *a* Obtained from (4,5)-7 and (8,7)-9 Calculations

		S_1_			S_2_		
		Contributions of transitions			Contributions of transitions		
	AS-roots	*E* (eV)	H → L, H – 1 → L + 1	|μ| (D)	*E* (eV)	H – 1 → L, H → L + 1	|μ| (D)
Chl *a*	(4,5)-7	1.988 (0.340)	0.73, 0.16	6.71	2.220 (0.004)	0.46, 0.40	0.72
	(8,7)-9	1.995 (0.266)	0.66, 0.16	5.93	2.279 (0.015)	0.42, 0.37	1.31
(Chl *a*)-His	(4,5)-7	1.960 (0.336)	0.72, 0.17	6.72	2.148 (0.004)	0.50, 0.38	0.69
	(8,7)-9	1.975 (0.257)	0.65, 0.18	5.85	2.244 (0.010)	0.44, 0.35	1.08
(Chl *a*)-H_2_O	(4,5)-7	1.994 (0.358)	0.72, 0.16	6.87	2.139 (0.006)	0.50, 0.36	0.86
	(8,7)-9	2.008 (0.271)	0.65, 0.16	5.96	2.231 (0.020)	0.45, 0.34	1.53
Pheo *a*	(4,5)-7	1.934 (0.272)	0.69, 0.20	6.09	2.370 (0.001)	0.46, 0.42	0.37
	(8,7)-9	1.932 (0.201)	0.62, 0.21	5.24	2.419 (0.005)	0.45, 0.38	0.75

To summarize, we present a specific CASSCF/NEVPT2 protocol for describing the low-energy region of the absorption spectra of all RC pigments. The (4,5) AS (Figure 4a) is the minimal AS that still allows meaningful and converged results to be obtained for the Q-band of Chl *a* and Pheo *a*, as well as axially substituted Chl *a*. However, since the description of higher excited states is not yet stable with respect to the AS, the reliable description of the B band remains an open challenge. In any case, this protocol can now be used to compute the site energies of protein-embedded chlorophyll and pheophytin pigments, therefore we can proceed with the application to the RC of PSII using a multiscale QM/MM approach to account for the effects of the protein environment.

### 3.2 Multireference Description of the PSII-RC

It is well-known that the protein environment significantly affects the excited state energies of photosynthetic pigments.^5−7,14,126−229^ For the RC of PSII, it has been established that the electrostatic effects of the protein matrix diversify the site energies and determine the nature of the primary processes, inducing excitation asymmetry between pigments of the D1 and D2 branches (Figure 2) and giving rise to unidirectional electron transfer via the D1 branch.^11^ Prior QM/MM studies using TD-DFT and DLPNO-STEOM-CCSD calculations suggested that the RC pigment with the lowest site energy under the influence of protein matrix effects is Chl_D1_. It is important to see whether this result still stands when the problem is approached with the current multireference CASSCF/NEVPT2 protocol combined with MM treatment of the protein matrix. To account for the influence of the protein environment on the structure of the pigments, geometry optimization of each pigment was carried out using multiscale QM/MM modeling. The inset figures included in Figure 2 depict the QM region defined for each pigment.

#### 3.2.1 Multireference QM/MM Description of Protein-Embedded RC Pigments

CASSCF/NEVPT2 calculations were performed on the QM/MM optimized geometry of each pigment, first without including the protein surroundings, i.e., in vacuo, and second, including the electrostatic environment of the protein using MM, denoted in protein. Comparison of the CASSCF(4,5)-7/NEVPT2 to the CASSCF(8,7)-9/NEVPT2 energies and oscillator strengths (Tables S10 and S11) show that the minimal AS determined in the previous sections for the gas-phase pigments is sufficient to describe the protein-embedded pigments as well. The CASSCF(4,5)-7/NEVPT2 site energies of all chlorophyll-based pigments are systematically slightly lower (by up to 0.022 eV) than the CASSCF(8,7)-9/NEVPT2 energies, both for the protein-embedded chromophores and in vacuum, whereas the CASSCF(4,5)-7/NEVPT2 site energies of the pheophytins are slightly higher (by 0.009 and 0.019 eV for Pheo_D1_ and Pheo_D2_, respectively) than the CASSCF(8,7)-9/NEVPT2 energies in vacuum, and lower (by 0.021 and 0.061 eV for Pheo_D1_ and Pheo_D2_) in protein.

**Figure 7:**
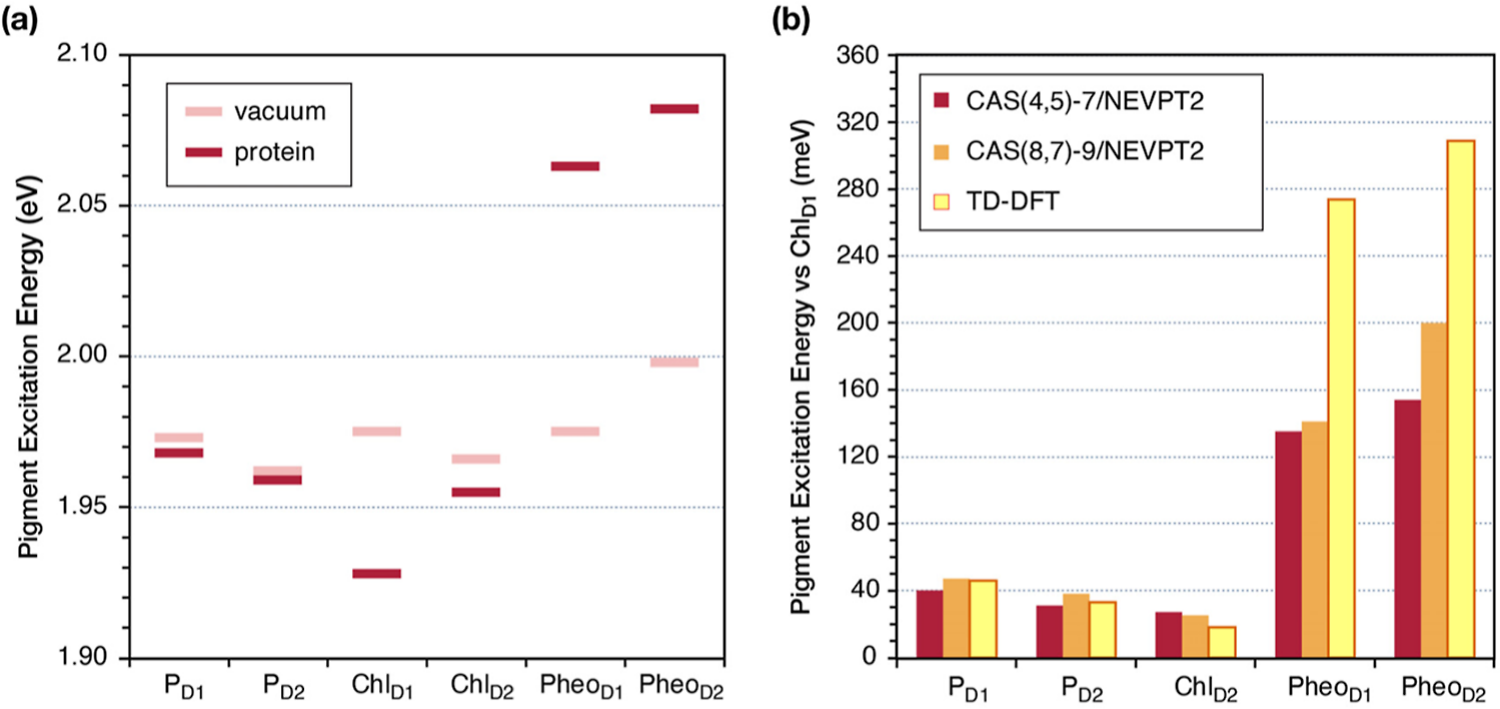
(a) Vertical excitation energy of the first excited state (Q_*y*_) of the individual RC pigments from CASSCF(4,5)-7/SC-NEVPT2 calculations *in vacuum* (light red) and from CASSCF(4,5)-7/SC-NEVPT2/MM calculations *in protein* (dark red). (b) Relative Q_*y*_ site energies of the individual RC pigments obtained with CASSCF(4,5)-7/SC-NEVPT2/MM (red), CASSCF(8,7)-9/SC-NEVPT2/MM (orange), and ωB97X-D3(BJ)/ΜΜ (yellow), referenced to the Chl_D1_ site energy.

The CASSCF(4,5)-7/NEVPT2 site energies of the six RC pigments calculated in vacuum (light red) and in protein (dark red) are plotted in Figure 7a. It can be observed that the site energies in vacuum are very similar, all being in the range between 1.962 and 1.998 eV. The protein matrix redshifts chlorophyll pigments, with Chl_D1_ featuring the largest red shift of 0.047 eV, whereas it strongly blueshifts the pheophytins. Notably, the nature and oscillator strengths of the two lowest excited states remain almost the same in the protein-embedded pigments as in vacuum (Table S10). The differences between the site energies in vacuum and in protein, i.e. the electrochromic shifts, reflect the protein electrostatic effects on the energies of the excited states. Site energies and electrochromic shifts obtained with our NEVPT2/MM protocol are compared with the corresponding results obtained from TD-DFT and STEOM-DLPNO-CCSD^11^ in Table S12. Overall, our CASSCF(4,5)-7/NEVPT2/MM protocol predicts that the protein-embedded Chl_D1_ exhibits the lowest and Pheo_D2_ the highest excitation energies, in agreement with single-reference methods.

Among the most important descriptors of the physical properties of the overall system are the relative site energies of the six pigments. The NEVPT2 and TD-DFT site energy differences of all pigments from Chl_D1_ are plotted in Figure 7b. All methods suggest that the site energies follow the sequence Pheo_D2_ > Pheo_D1_ > P_D1_ > P_D2_ > Chl_D2_ > Chl_D1_ (see also Tables S13–S15). In view of the present results, it is likely that TD-DFT (Figure 7b, yellow bars) exaggerated the difference between chlorophyll and pheophytin site energies,^11^ predicting an even more pronounced transverse asymmetry in the RC. Overall, results from a spectrum of methodologies, spanning from TD-DFT to coupled cluster and ultimately to multireference wave function-based approaches, collectively delineate the influence of the protein matrix on the D1/D2 excitation asymmetry. This indicates the reliability of the computational methodologies employed but also strengthens the conclusions drawn concerning the properties and function of the RC, confirming that the D1 versus D2 branch excitation asymmetry is induced by the electrostatic effect of the protein matrix.

#### 3.2.2 Effect of Geometry

Having established a reliable multireference-multilevel approach to calculate the vertical excitation energies, we address a primary methodological choice that is the geometry optimization method. In the previous section, we presented the results using models where the corresponding pigment (QM region) was optimized with DFT and the rest of the protein, surrounding lipids, and crystallographic water molecules (MM region) were equilibrated with MD, as described in the Computational Details section. These models are denoted **C** (QM|MD) and are the most realistic representation achievable for the system in physiological conditions.

We first examine the importance of geometry optimization of each pigment, and, second, the effect of the protein matrix equilibration. To this end, we compare the site energies calculated with models **C** with those of models **B** (QM|XRD), where the whole pigment (QM region) was optimized with DFT, whereas all heavy atoms of the MM region were kept in their crystallographic (XRD) positions, and with those of models **A** (XRD|XRD), where all heavy atoms of both the QM and MM regions were kept in their XRD positions (i.e., only the H atoms were optimized).

**Figure 8:**
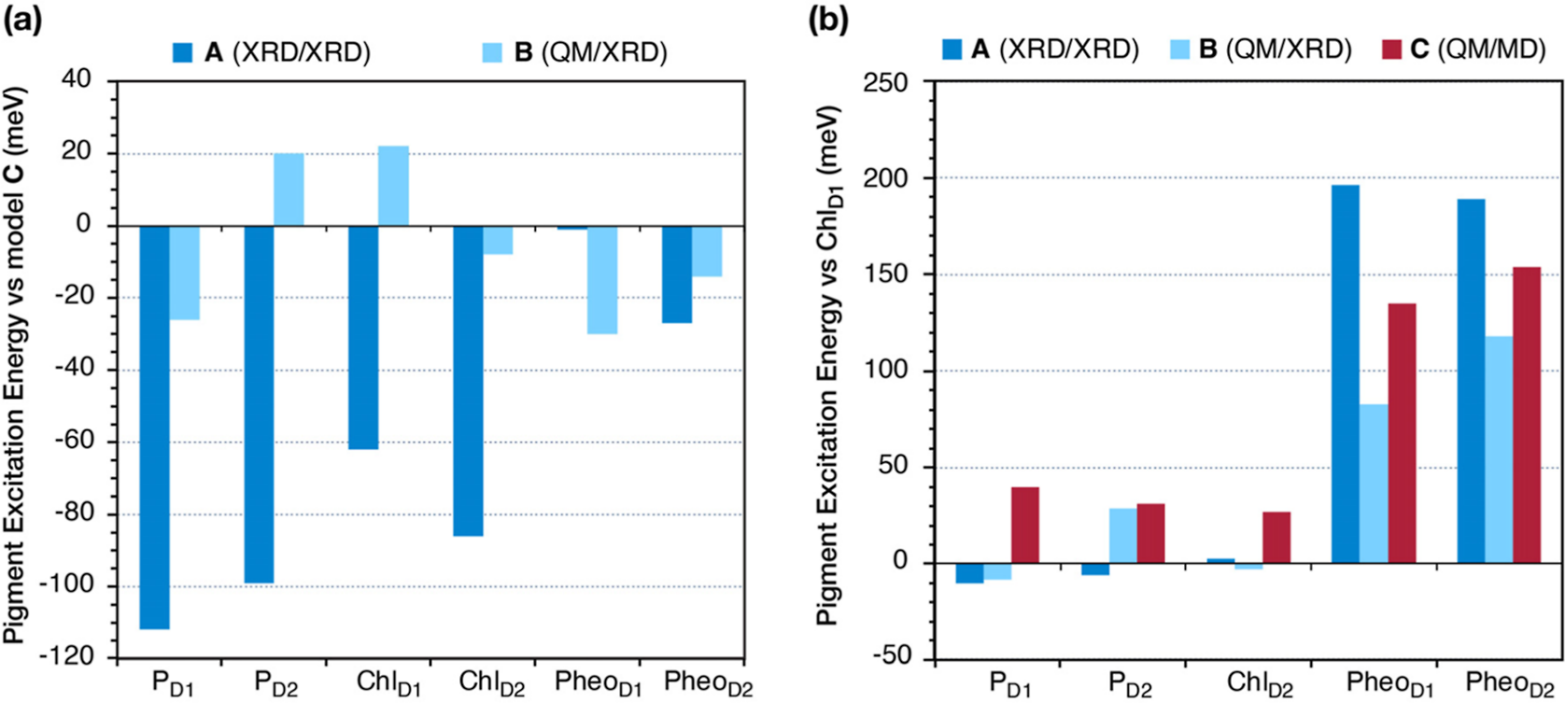
(a) Deviations of the CASSCF(4,5)-7/NEVPT2 site energies obtained from the **A** (XRD|XRD) (dark blue) and the **B** (QM|XRD) (light blue) models from those obtained from the **C** (QM|MD) models. (b) Relative site energies of the RC pigments obtained with models type **A**, **B**, and **C**, referenced to the Chl_D1_ site energy.

In Figure 8a, the site energy differences of models **A** and **B** from model **C** and plotted (extensive results given in Table S15). The excitation energy differences between models **B** and **C** are relatively small, less than 0.03 eV. By contrast, larger differences, up to 0.12 eV, are observed between **A** and **B** (as well as between **A** and **C**), especially for chlorophyll-based pigments. Most importantly, Figure 8b shows remarkable differences in the relative excitation energies among the six pigments for models **A**, **B**, and **C**. The pigment with the lowest site energy in the RC is predicted to be P_D1_ for both type **A** and type **B** models, instead of Chl_D1_ predicted using model type **C**. It is important to note that similar results regarding the relative site energies of the six pigments using **A**, **B**, and **C** models are obtained with TD-DFT (Table S13). Interestingly, P_D1_ was predicted to be the chlorophyll with the lowest site energy of the RC in a recent study by Sørensen et al.,^63^ where the calculations were performed on the same crystallographic coordinates we use in this work (3WU2). Therefore, using the crystallographic coordinates without geometry optimization leads to fundamentally different conclusions about the system. A plausible explanation for this erroneous description obtained using XRD pigment geometries is the well-recognized inaccuracy of low-to-medium resolution crystallographic structures to identify the bond length alternation (BLA) pattern of conjugated systems.^129,130^ Therefore, the present results highlight the importance of refining the crystallographic structures, both for the pigments and for the protein matrix, as also previously emphasized by Dreuw et al.^129^

A final note concerns the optimal QM method for geometry optimization. Generally, the Hartree–Fock method tends to overestimate the BLA of conjugated systems, whereas pure DFT methods tend to underestimate it. This general trend applies perfectly to the case of Chl *a*, as shown in Figure S9. Chl *a* models optimized with nonstandard PBE0 functional with lower % HF exchange feature lower BLA values and their excitation energies are predicted to be systematically lower [∼0.09 eV difference for the S_0_ → S_1_ excitation for Chl *a* optimized with PBE0 (25% HF) and PBE (0% HF)]. At this point we cannot determine the objectively best method for geometry optimization; this will be the subject of an upcoming benchmark study. In any case, the excitation energy differences are *systematic*, which is confirmed by the TD-DFT electrochromic shifts on the PBE0 and PBE optimized RC pigments which are very similar (Tables S13 and S14).

## 4 Conclusions and Perspectives

In this study we have developed a multireference-multilevel computational protocol for the description of the lowest excited states (Q-band) of protein-embedded chlorophyll and pheophytin pigments. We suggest a transparent and precise SA-CASSCF/NEVPT2 protocol, derived from systematic exploration of the different methodological choices involved in the SA-CASSCF description of Chl *a* excited states. First, we describe how the AS and number of roots included in the SA-CASSCF orbital optimization are interconnected. Specifically, irrespective of the AS, at least five states, i.e. the ground state and four excited states, should be included in the SA-CASSCF orbital optimization in order to obtain stable and meaningful results for the Q-band using NEVPT2. Although the absolutely minimal Gouterman space, i.e., the four frontier orbitals (4,4) AS, retains its validity in providing a qualitative picture that is useful as a conceptual aid, we show that it is strictly inadequate for reproducing the S_1_–S_2_ energy difference. The minimal AS that correctly predicts not only the nature but also the energies of the Q-band excited states is the (4,5) AS with 7 roots, i.e. we suggest a CASSCF(4,5)-7/NEVPT2 as the absolute minimal approach for a reliable description of the Q-band.

Crucially, we find that the balanced expansion of the AS beyond this point is not obvious but should be chemically guided, taking into account the orbital symmetries, the nature of the Q-band transitions, and the stability of the results with respect to the number of roots included in the SA orbital optimization. Our systematic investigation showed that after (4,5)-7, the immediately larger AS is (8,7) with 9 roots. This AS results from rotating two lower-energy occupied orbitals (H – 5 and H – 6) into the AS and not from a simplistic energy-based expansion. The proposed (8,7) AS provides excellent agreement with available experimental data for the gas-phase Chl *a*, not only for the Q-band but also for the B band. The next step is the (14,11) AS, which already exhausts all room for improvement of the Q-band states. The Q-band description obtained from the (8,7)-9 AS is the same as that of the (14,11)-10 AS, which suggests that the CASSCF description is converged with respect to the size of the AS and any further expansion might compromise the accuracy of the Q-band results. We note, however, that the CASSCF description of the B band is still not stable, and further investigation is necessary to extend our protocol rationally in this direction.

Importantly, we find that the same requirements apply for axially substituted Chl *a* molecules and for pheophytin, which makes the protocol applicable to all RC pigments of PSII. Application of the CASSCF/NEVPT2 protocol using a multilevel QM/MM approach to compute the site energies of pigments in the RC of PSII confirms that the protocol is eminently applicable in a realistic large-scale context. The results reaffirm the protein matrix-induced excitation asymmetry (both lateral and transverse) that was previously found for the PSII RC using single-reference QM/MM approaches. We also find that the protein effects uniquely diversify the absorption properties of the six pigments, not by altering the geometry of the pigments, but through electrostatic interactions with the surrounding protein matrix, rendering Chl_D1_ the pigment with the lowest site energy. Crucially, the present NEVPT2/MM results provide a more moderate transverse asymmetry compared to TD-DFT calculations, potentially correcting a pronounced blue-shift of pheophytins relative to chlorophylls reported in previous multiscale simulations of the RC.^11^ The overall consensus between fundamentally different computational methodologies reinforces confidence with respect to the robustness of the methodology and the physical properties of the RC.^6^

Having established a reliable protocol for multiscale simulations of the Q-band states of protein-embedded single pigments, in future studies we will explore extensions to the description of the Soret band and of higher energy excitations, as well as for the description of excited states of multiple pigments. This will allow the multireference QM/MM treatment of coherent excitation, charge transfer, and charge separation in pigment assemblies. Moreover, it will provide the basis for multireference ab initio dynamics approaches that will leverage recent developments in the efficient treatment of very large ASs.

## Supporting Information Available

CASSCF(4,4) and NEVPT2 results for Chl *a* (Tables S1–S3 and Figure S1); CASSCF/NEVPT2 results from expanded ASs (Tables S4–S8); CASSCF optimized orbitals and average orbital occupations (Figures S2–S5); comparison of CASSCF and NEVPT2 excitation energies for the (14,11) AS (Figure S6); comparison of SC-NEVPT2 to FIC-NEVPT2, and triple-ζ to quadruple-ζ basis sets (Table S9 and Figure S7); results for Chl *a* axially substituted with His and H_2_O, and for Pheo *a* (Figure S8); CASSCF/NEVPT2/MM results for PSII RC pigments in vacuum and in protein (Tables S10–S11); comparison of CASSCF/NEVPT2/MM results with TD-DFT/MM and DLPNO-STEO-CCSD/MM (Table S12); ωB97X-D3(BJ) TD-DFT results for models of PSII RC pigments optimized with the PBE0 and PBE functionals (Tables S13–S14); comparison of results for geometries **A** (XRD|XRD), **B** (QM|XRD), and **C** (QM|MD) (Table S15); results for Chl *a* optimized with different % HF exchange, and correlation between the BLA of Chl *a* and the % HF exchange (Figure S9) (PDF)
Cartesian coordinates of all structural models discussed in this work (TXT)
Complete numerical results of CASSCF and NEVPT2 calculations for all models (TXT)
