# Supplementary material for: Combined Multireference–Multiscale Approach to the Description of Photosynthetic Reaction Centers
Source: J Chem Theory Comput. 2024 Aug 8;20(16):7210–26. doi: 10.1021/acs.jctc.4c00578 (PMC11360140; doi:10.1021/acs.jctc.4c00578)
Supplement: Supplementary file 1 — ct4c00578_si_001.pdf [file ct4c00578_si_001.pdf]

SUPPORTING INFORMATION

for

Combined Multireference–Multiscale Approach to the Description of Photosynthetic Reaction Centers

Maria Drosou, Sinjini Bhattacharjee, Dimitrios A. Pantazis

Table S1. CASSCF(4,4) vertical excitation energies (in eV) for Chl *a* depending on the number of excited states (1–8) included in the state-averaged orbital optimization. Oscillator strengths are indicated in parentheses.

Number of roots	S_1	S_2	S_3	S_4	S_5	S_6	S_7	S_8
2	2.991 (0.580)							
3	3.083 (0.151)	3.376 (0.918)						
4	3.065 (0.678)	3.397 (0.002)	4.739 (1.589)					
5	3.013 (0.689)	3.445 (0.005)	4.663 (1.568)	4.989 (1.370)				
6	3.117 (0.639)	3.381 (0.014)	4.946 (1.628)	5.254 (1.433)	5.473 (0.634)			
7	3.109 (0.530)	3.450 (0.006)	5.004 (1.489)	5.275 (1.596)	5.506 (0.912)	5.874 (0.519)		
8	3.126 (0.502)	3.469 (0.006)	5.076 (1.416)	5.323 (1.558)	5.481 (1.081)	5.821 (0.567)	6.723 (0.018)	
9	3.149 (0.533)	3.458 (0.008)	5.070 (1.363)	5.344 (1.369)	5.429 (1.154)	5.870 (0.727)	6.722 (0.013)	7.087 (0.007)

Table S2. NEVPT2 vertical excitation energies (in eV) obtained from SA-CASSCF(4,4) calculations depending on the number of roots (2–9) included in the state-averaged orbital optimization. Oscillator strengths are indicated in parentheses. NEVPT2 often results in reordering of the CASSCF states. The numbers in brackets indicate the corresponding CASSCF excited state with respect to the excited states listed in Table S1.

Number of roots	S_1	S_2	S_3	S_4	S_5	S_6	S_7	S_8
2	2.053<br>(0.398)<br>[1]							
3	1.870<br>(0.508)<br>[2]	2.473<br>(0.121)<br>[1]						
4	1.976<br>(0.437)<br>[1]	2.126<br>(0.001)<br>[2]	2.802<br>(0.939)<br>[3]					
5	2.017<br>(0.461)<br>[1]	2.118<br>(0.003)<br>[2]	2.878<br>(0.968)<br>[3]	3.018<br>(0.829)<br>[4]				
6	1.938<br>(0.397)<br>[1]	2.087<br>(0.009)<br>[2]	2.777<br>(0.914)<br>[3]	2.829<br>(0.772)<br>[4]	3.143<br>(0.364)<br>[5]			
7	1.933<br>(0.330)<br>[1]	2.073<br>(0.003)<br>[2]	2.861<br>(0.865)<br>[4]	2.874<br>(0.855)<br>[3]	3.143<br>(0.278)<br>[6]	3.178<br>(0.526)<br>[5]		
8	1.920<br>(0.308)<br>[1]	2.068<br>(0.004)<br>[2]	2.900<br>(0.849)<br>[4]	2.934<br>(0.819)<br>[3]	3.143<br>(0.620)<br>[5]	3.203<br>(0.312)<br>[6]	3.990<br>(0.011)<br>[7]	
9	1.902<br>(0.322)<br>[1]	2.065<br>(0.005)<br>[2]	2.939<br>(0.790)<br>[3]	2.987<br>(0.765)<br>[4]	3.055<br>(0.649)<br>[5]	3.173<br>(0.393)<br>[6]	3.697<br>(0.004)<br>[8]	4.000<br>(0.008)<br>[7]

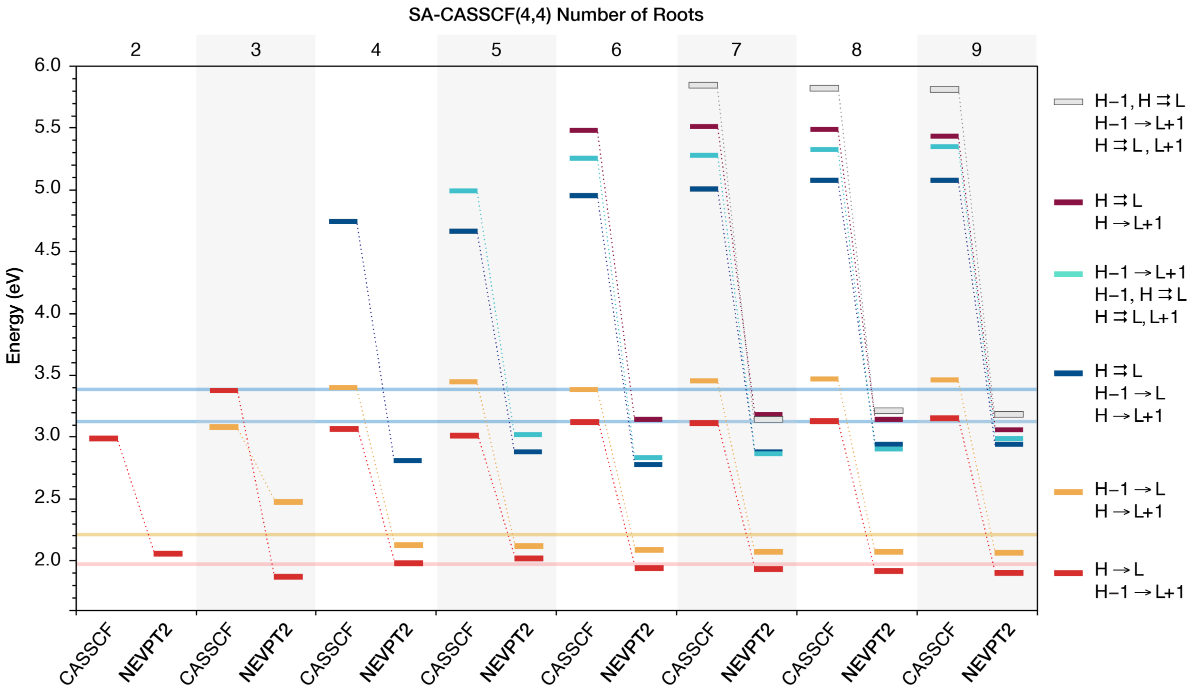

Figure S1. Comparison of CASSCF and NEVPT2 vertical excitation energies for Chl *a* derived from SA-CASSCF(4,4) calculations depending on the number of roots (2–9) included in the state-averaged orbital optimization.

Table S3. NEVPT2 excitation energies (in eV) obtained from SA-CASSCF(4,4) calculations depending on the number of roots (2–9) included in the state-averaged orbital optimization. Calculated oscillator strengths are indicated in parentheses. The state contributions of the leading configurations and the corresponding transition dipole moments (in Debye) are also given.

	S_1			S_2		
Number of roots	*E*	Contributions of transitions: H→L, H−1→L+1	|*μ*|	*E*	Contributions of transitions: H−1→L, H→L+1	|*μ*|
2	2.053 (0.398)	0.81, 0.12	7.14			
3	1.870 (0.508)	0.74, 0.06	8.46	2.473 (0.121)	0.32, 0.28	3.59
4	1.976 (0.437)	0.78, 0.14	7.63	2.126 (0.001)	0.48, 0.39	0.39
5	2.017 (0.461)	0.74, 0.13	7.76	2.118 (0.003)	0.48, 0.36	0.63
6	1.938 (0.397)	0.76, 0.12	7.35	2.087 (0.009)	0.41, 0.39	1.05
7	1.933 (0.330)	0.76, 0.15	6.70	2.073 (0.003)	0.47, 0.40	0.65
8	1.920 (0.308)	0.76, 0.16	6.50	2.068 (0.004)	0.50, 0.43	0.68
9	1.902 (0.322)	0.76, 0.15	6.68	2.065 (0.005)	0.48, 0.43	0.76

Table S4. NEVPT2 excitation energies (in eV) obtained from SA-CASSCF(4,5) calculations depending on the number of roots (2–9) included in the state-averaged orbital optimization. Calculated oscillator strengths are indicated in parentheses. The state contributions of the leading configurations and the corresponding transition dipole moments (in Debye) are also given.

	S_1			S_2		
Number of roots	*E*	Contributions of transitions: H→L, H−1→L+1	$\|\mu\|$	*E*	Contributions of transitions: H−1→L, H→L+1	$\|\mu\|$
2	2.208 (0.565)	0.82, 0.08	8.21			
3	2.123 (0.203)	0.46, 0.06	5.02	2.198 (0.239)	0.19, 0.26	5.35
4	2.120 (0.623)	0.75, 0.05	8.80	2.564 (0.088)	0.37, 0.37	3.00
5	2.011 (0.361)	0.72, 0.13	6.88	2.230 (0.007)	0.46, 0.38	0.89
6	1.999 (0.360)	0.72, 0.14	6.88	2.215 (0.011)	0.42, 0.39	1.14
7	1.988 (0.340)	0.73, 0.16	6.71	2.220 (0.004)	0.46, 0.40	0.72
8	1.985 (0.341)	0.73, 0.16	6.72	2.215 (0.002)	0.46, 0.40	0.50
9	1.982 (0.331)	0.71, 0.16	6.63	2.211 (0.004)	0.46, 0.40	0.66

Table S5. NEVPT2 excitation energies (in eV) obtained from SA-CASSCF(4,6) calculations depending on the number of roots (2–9) included in the state-averaged orbital optimization. Calculated oscillator strengths are indicated in parentheses. The state contributions of the leading configurations and the corresponding transition dipole moments (in Debye) are also given.

	S_1			S_2		
Number of roots	*E*	Contributions of transitions: H→L, H−1→L+1	$\|\mu\|$	*E*	Contributions of transitions: H−1→L, H→L+1	$\|\mu\|$
2	2.128 (0.423)	0.79, 0.11	7.23			
3	2.166 (0.310)	0.59, 0.08	6.14	2.273 (0.147)	0.26, 0.33	4.12
4	2.087 (0.451)	0.72, 0.10	7.54	2.475 (0.028)	0.45, 0.36	1.73
5	2.036 (0.341)	0.72, 0.14	6.64	2.308 (0.003)	0.45, 0.39	0.59
6	2.027 (0.363)	0.71, 0.15	6.87	2.321 (0.004)	0.42, 0.41	0.71
7	2.022 (0.316)	0.71, 0.17	6.41	2.288 (0.003)	0.45, 0.42	0.58
8	2.023 (0.392)	0.74, 0.14	7.15	2.224 (0.004)	0.47, 0.39	0.69
9	2.021 (0.379)	0.71, 0.15	7.02	2.224 (0.004)	0.47, 0.38	0.72

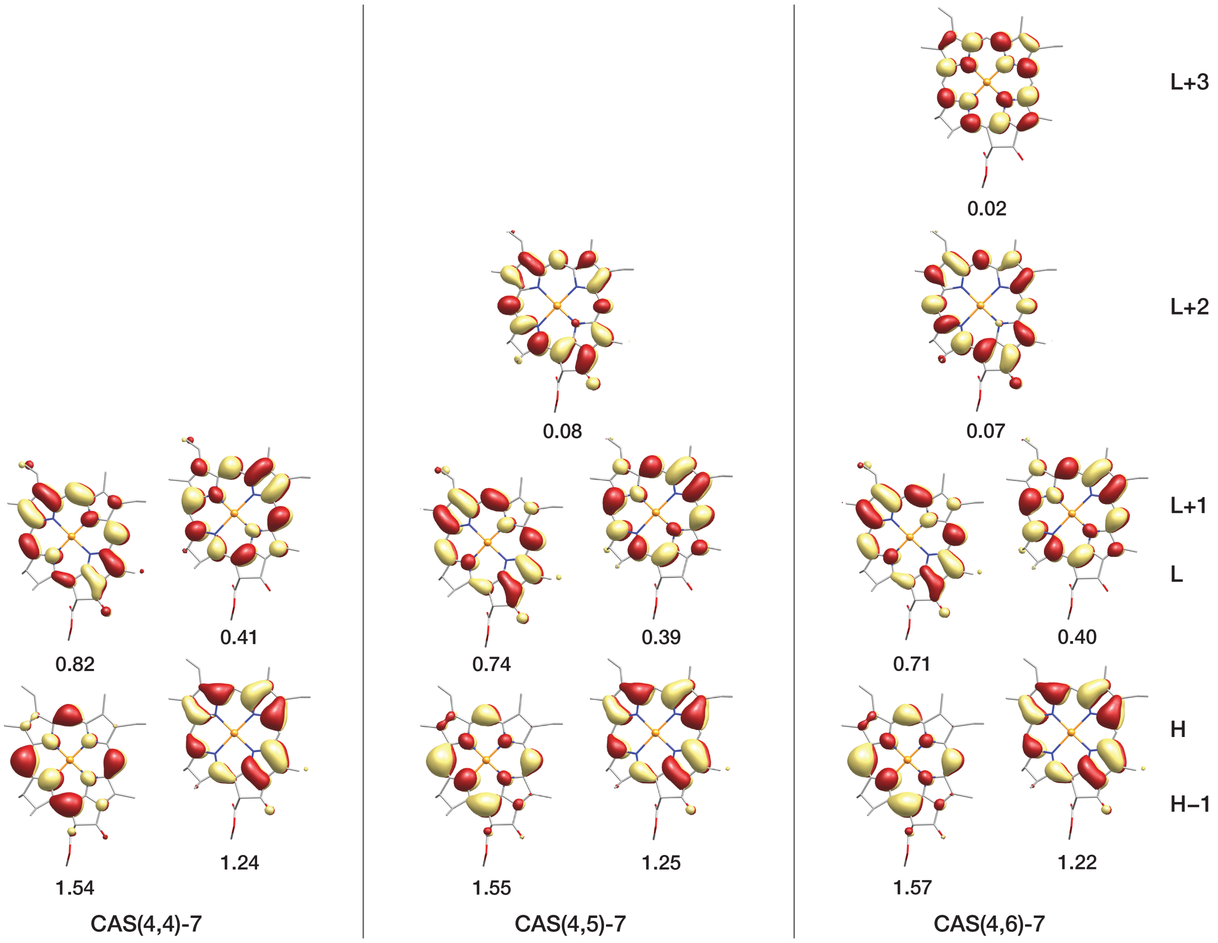

Figure S2. Optimized orbitals and average orbital occupations from the (4,4)-7, (4,5)-7, and (4,6)-7 calculations.

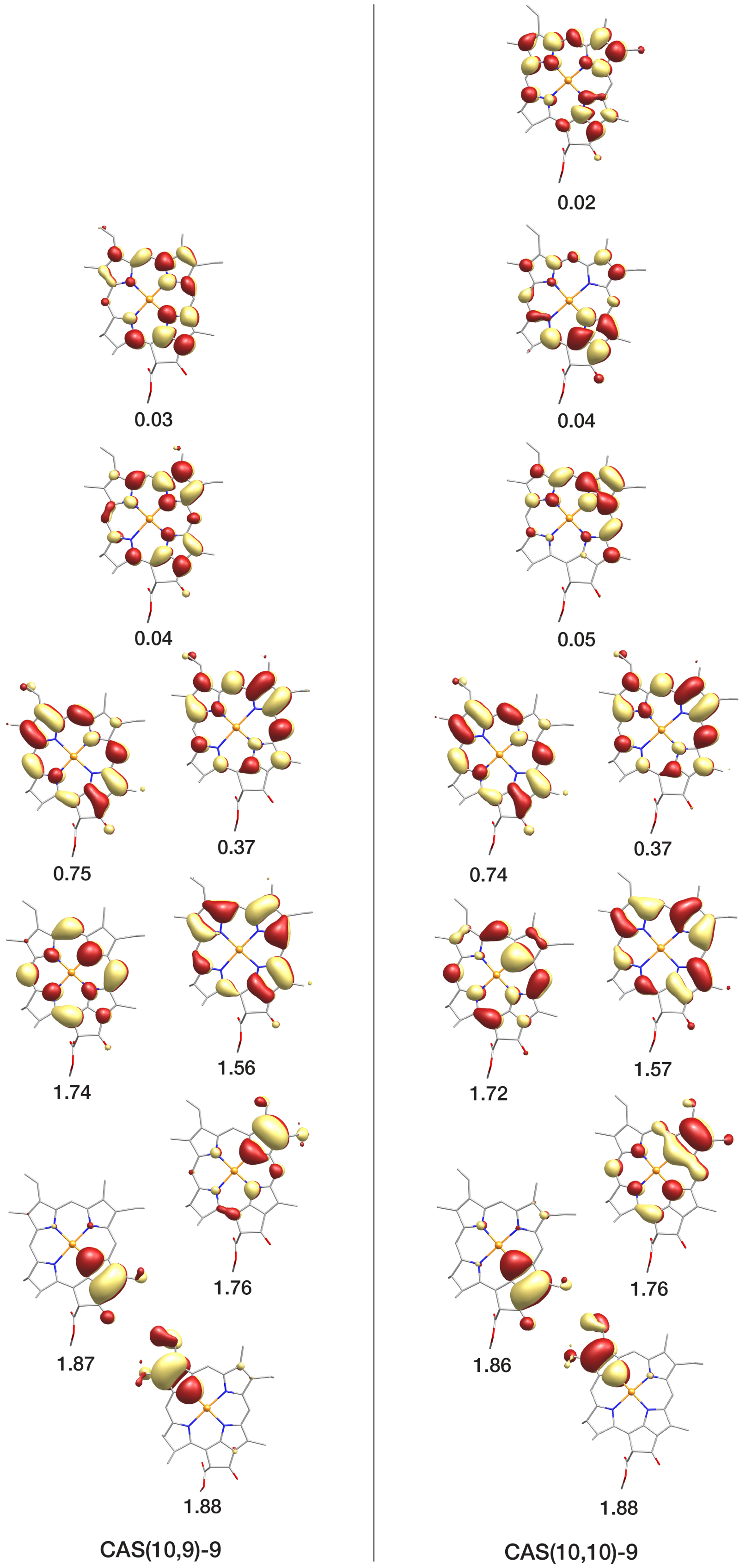

Figure S3. Optimized orbitals and average orbital occupations from the (10,9)-9 and (10,10)-9 calculations.

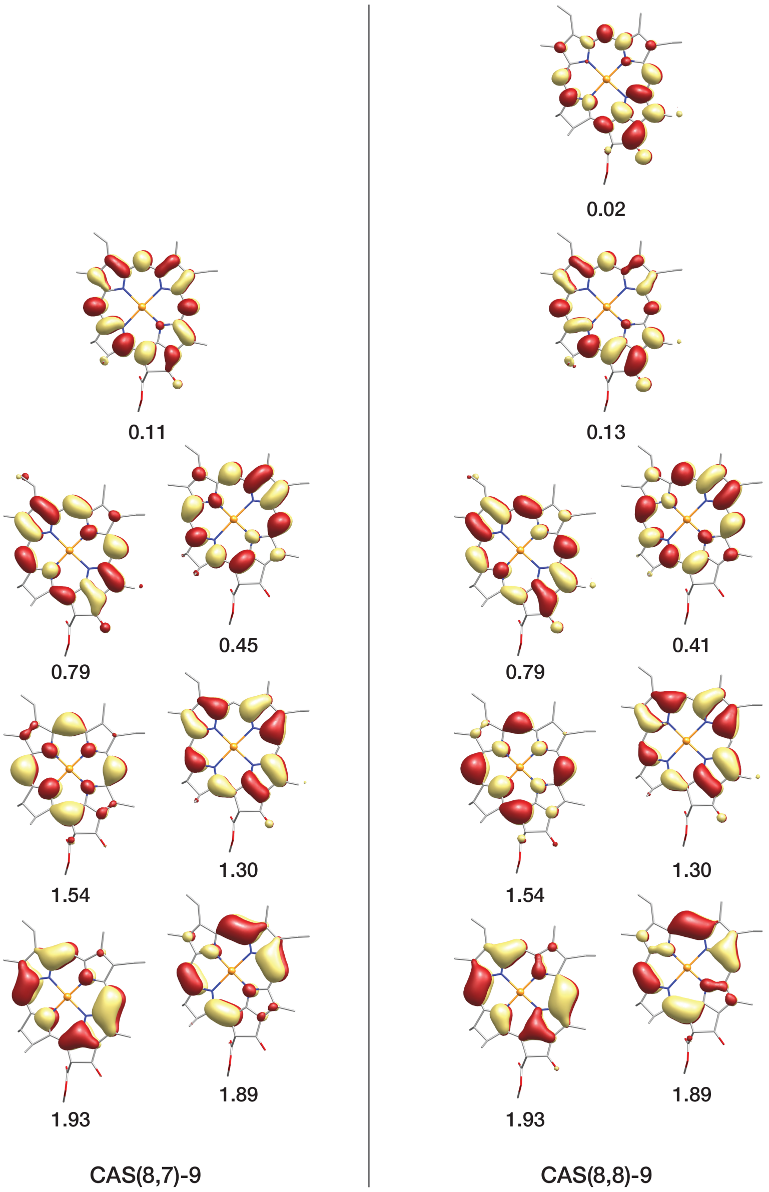

Figure S4. Optimized orbitals and average orbital occupations from the (8,7)-9 and (8,8)-9 calculations.

Table S6. NEVPT2 excitation energies (in eV) obtained from SA-CASSCF(8,7) calculations depending on the number of roots (2–10) included in the state-averaged orbital optimization. Calculated oscillator strengths are indicated in parentheses. The state contributions of the leading configurations and the corresponding transition dipole moments (in Debye) are also given.

	S_1			S_2		
Number of roots	*E*	Contributions of transitions: H→L, H−1→L+1	|*μ*|	*E*	Contributions of transitions: H−1→L, H→L+1	|*μ*|
2	2.327 (0.616)	0.76, 0.04	8.35			
3	2.140 (0.353)	0.68, 0.11	6.59	2.351 (0.033)	0.35, 0.34	1.93
4	2.066 (0.237)	0.56, 0.08	5.49	2.267 (0.147)	0.34, 0.19	4.14
5	1.984 (0.290)	0.66, 0.13	6.21	2.301 (0.015)	0.40, 0.33	1.32
6	1.950 (0.257)	0.67, 0.16	5.89	2.294 (0.007)	0.44, 0.37	0.88
7	1.966 (0.276)	0.68, 0.16	6.08	2.316 (0.002)	0.41, 0.38	0.50
8	2.004 (0.276)	0.65, 0.15	6.02	2.302 (0.013)	0.40, 0.35	1.22
9	1.995 (0.266)	0.66, 0.16	5.93	2.279 (0.015)	0.42, 0.37	1.31
10	2.006 (0.263)	0.65, 0.17	5.88	2.270 (0.024)	0.44, 0.36	1.66

Table S7. NEVPT2 excitation energies (in eV) obtained from SA-CASSCF(14,11) calculations depending on the number of roots (8–10) included in the state-averaged orbital optimization. Oscillator strengths are indicated in parentheses. The state contributions of the leading configurations and the corresponding transition dipole moments (in Debye) are also given.

	S_1			S_2		
Number of roots	*E*	Contributions of transitions: H→L, H−1→L+1	|*μ*|	*E*	Contributions of transitions: H−1→L, H→L+1	|*μ*|
8	2.073 (0.360)	0.68, 0.13	6.77	2.382 (0.023)	0.42, 0.31	1.59
9	2.061 (0.348)	0.65, 0.13	6.67	2.373 (0.016)	0.39, 0.34	1.33
10	2.035 (0.300)	0.67, 0.14	6.23	2.341 (0.004)	0.40, 0.35	0.71

Table S8. NEVPT2 excitation energies (in eV) obtained from SA-CASSCF(16,12) and (16,13) calculations with 9 excited states included in the state-averaged orbital optimization. Oscillator strengths are indicated in parentheses. The state contributions of the leading configurations and the corresponding transition dipole moments (in Debye) are also given.

	S_1			S_2		
AS-roots	*E*	Contributions of transitions: H→L, H−1→L+1	|*μ*|	*E*	Contributions of transitions: H−1→L, H→L+1	|*μ*|
(16,12)-10	2.145 (0.296)	0.65, 0.15	6.03	2.374 (0.021)	0.42, 0.33	1.54
(16,13)-10	2.119 (0.310)	0.61, 0.14	6.21	2.381 (0.004)	0.38, 0.32	0.67

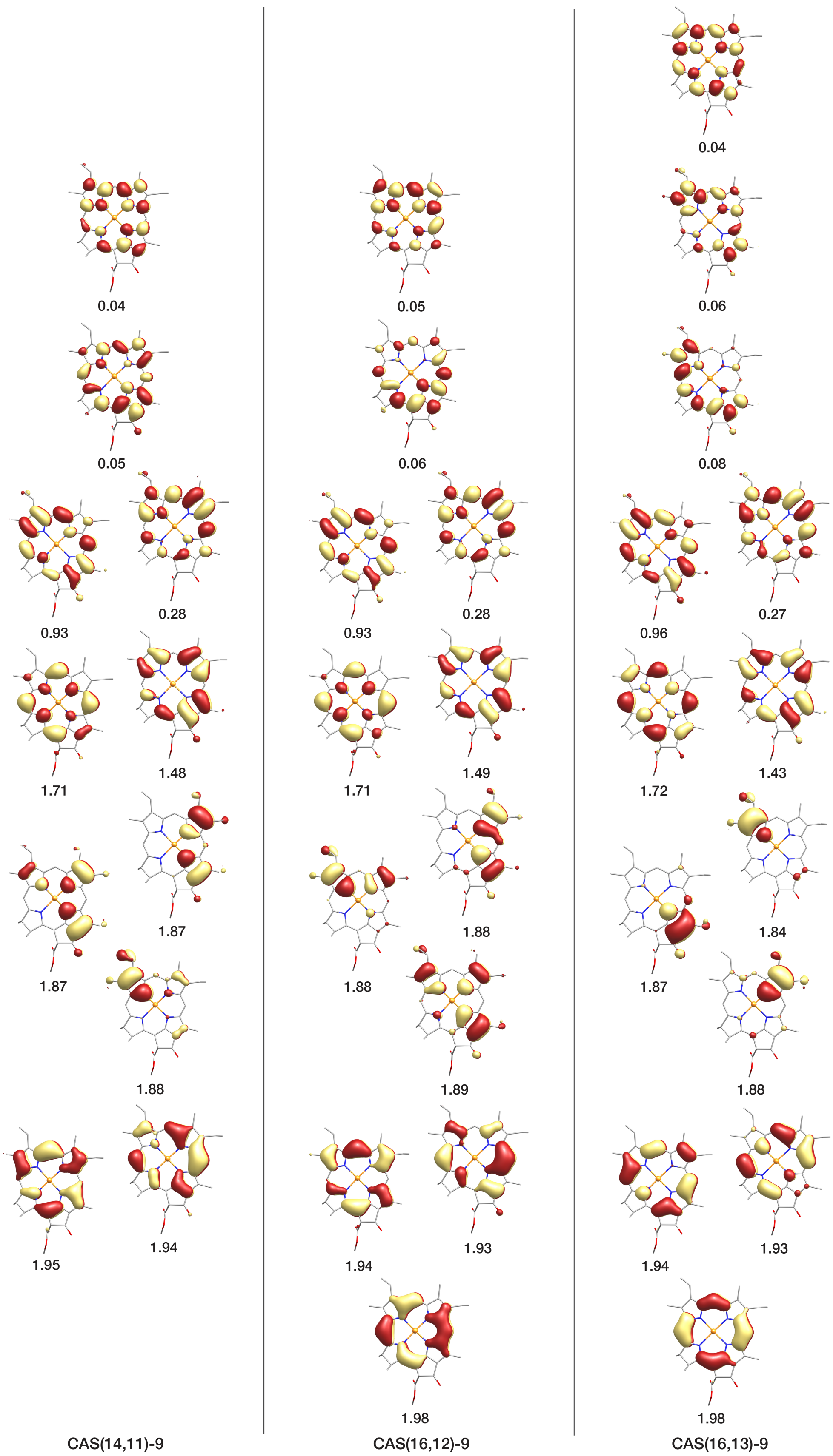

Figure S5. Optimized orbitals and average orbital occupations from the (14,11)-9, (16,12)-9, and (16,13)-9 calculations.

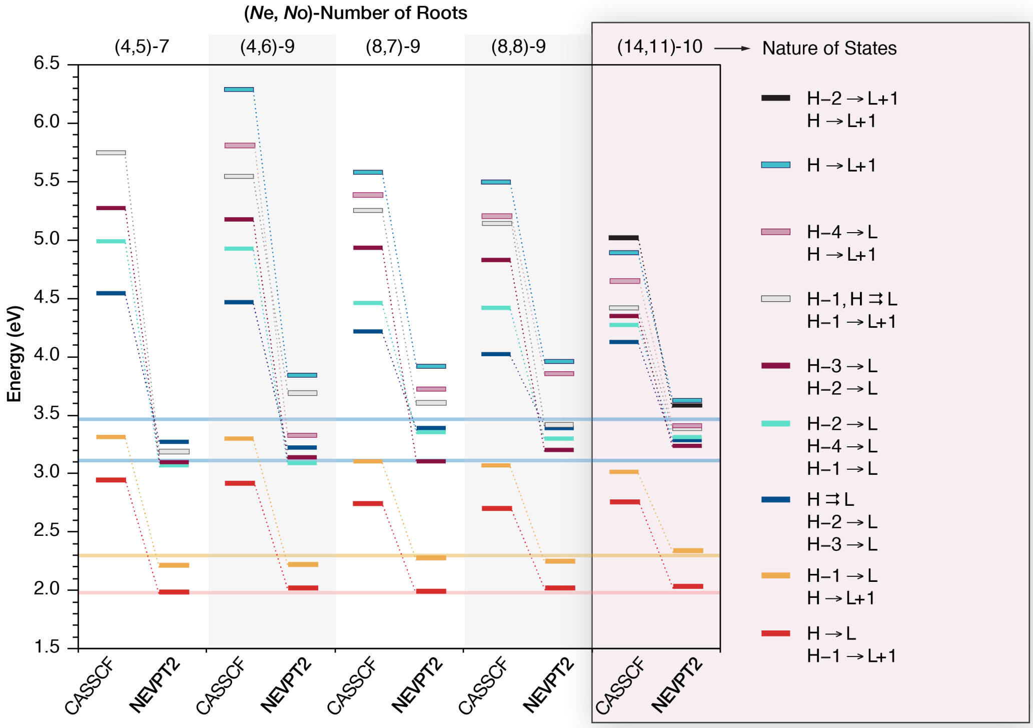

Figure S6. Comparison of CASSCF and NEVPT2 excitation energies for Chl *a* derived from SA-CASSCF calculations with different ASs and number of roots. The nature of S_1 and S_2 states is similar for all ASs (as shown in Figure 4d of the main text), whereas the nature of the higher excited states is different. The transitions with the higher (>10%) contributions on all excited states derived from the (14,11)-10 calculation are given in the shaded box.

Table S9. Excitation energies (eV) of Chl *a* derived from SC-NEVPT2 with def2-TZVP (TZ) and with def2-QZVPP (QZ) basis sets, and from FIC-NEVPT2 (with the def2-TZVP basis sets) using the (4,5)-7 and (8,7)-9 calculations.

AS-roots		S_1	S_2	S_3	S_4	S_5	S_6	S_7	S_8
(4,5)-7	SC-NEVPT2, TZ	1.988	2.220	3.081	3.096	3.191	3.272		
	SC-NEVPT2, QZ	1.969	2.201	3.054	3.060	3.160	3.243		
	FIC-NEVPT2, TZ	1.925	2.154	2.929	2.931	3.022	3.129		
(8,7)-9	SC-NEVPT2, TZ	1.995	2.279	3.102	3.351	3.388	3.605	3.795	3.918
	SC-NEVPT2, QZ	1.977	2.265	3.071	3.332	3.367	3.553	3.792	3.888
	FIC-NEVPT2, TZ	1.940	2.225	2.940	3.262	3.298	3.433	3.651	3.741

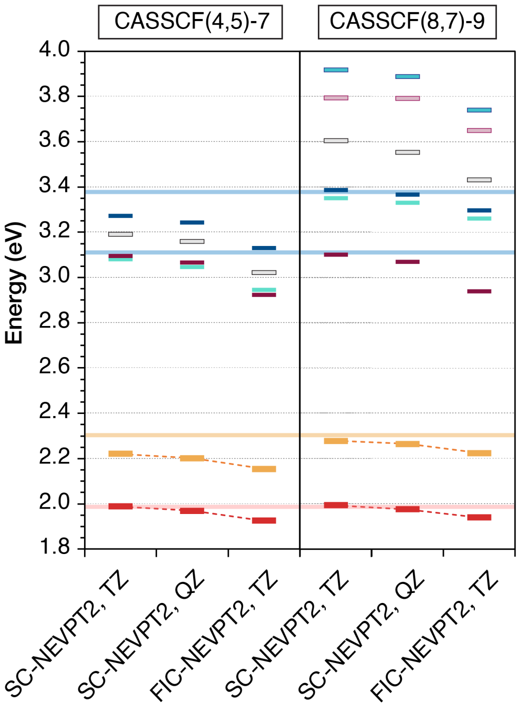

Figure S7. Comparison of excitation energies of Chl *a* derived from SC-NEVPT2 with def2-TZVP (TZ) and with def2-QZVP (QZ) basis sets, and from FIC-NEVPT2 (def2-TZVP basis sets) using the (4,5)-7 and (8,7)-9 calculations. Each color corresponds to the same CASSCF root of the wavefunction, but the nature of the states that are higher than the S_2 state is different between the (4,5)-7 and (8,7)-9 calculations.

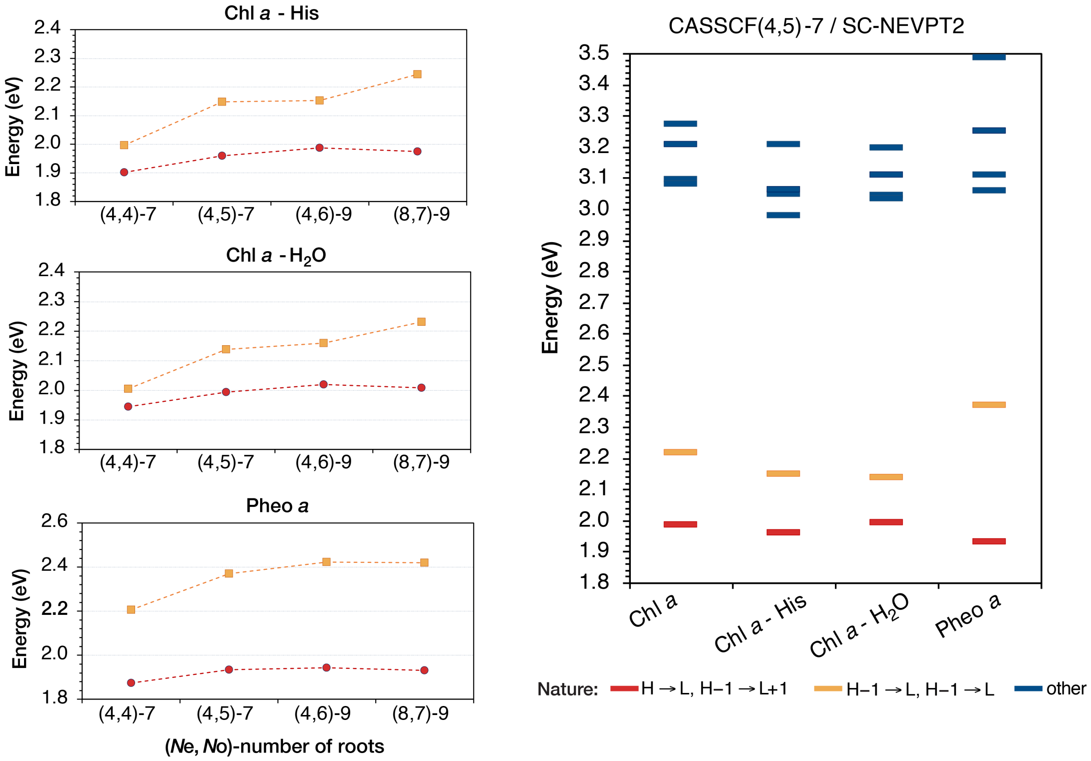

Figure S8. NEVPT2 excitation energies of gas-phase optimized models of Chl *a* axially substituted with histidine (His) and H_2O, and of Pheo *a*: (left) S_1 and S_2 excited states using different AS and number of roots and (right) all computed excited states using the (4,5)-7 AS.

Table S10. NEVPT2 energies (in eV) of the first (S_1) and second (S_2) excited states of PSII reaction center pigments in vacuum and in the protein electrostatic environment, using QM/MM optimized geometries for each pigment, i.e. geometries **C** (QM | MD), as defined in the computational details. The calculations were performed with the SA-CASSCF(4,5) calculations with 7 roots included in the state-averaged orbital optimization. Oscillator strengths are indicated in parentheses. The state contributions of the leading configurations and the corresponding transition dipole moments (in Debye) are also given.

		S_1			S_2		
			Contributions of transitions:			Contributions of transitions:	
		E	H→L, H−1→L+1	|*μ*|	*E*	H−1→L, H→L+1	|*μ*|
P_{D1}	vacuum	1.973 (0.344)	0.72, 0.17	6.77	2.151 (0.005)	0.53, 0.35	0.79
	protein	1.968 (0.379)	0.75, 0.16	7.12	2.178 (0.005)	0.54, 0.35	0.78
P_{D2}	vacuum	1.962 (0.326)	0.73, 0.17	6.61	2.148 (0.004)	0.50, 0.38	0.70
	protein	1.959 (0.352)	0.74, 0.16	6.87	2.178 (0.006)	0.50, 0.38	0.84
Chl_{D1}	vacuum	1.975 (0.354)	0.73, 0.16	6.87	2.138 (0.005)	0.52, 0.36	0.79
	protein	1.928 (0.409)	0.75, 0.15	7.47	2.137 (0.009)	0.56, 0.33	1.03
Chl_{D2}	vacuum	1.966 (0.340)	0.73, 0.17	6.75	2.165 (0.003)	0.50, 0.38	0.64
	protein	1.955 (0.360)	0.74, 0.16	6.96	2.191 (0.002)	0.50, 0.39	0.53
$Pheo_{D1}$	vacuum	1.975 (0.274)	0.68, 0.19	6.05	2.362 (0.001)	0.48, 0.40	0.32
	protein	2.063 (0.290)	0.67, 0.18	6.09	2.285 (0.007)	0.53, 0.34	0.93
$Pheo_{D2}$	vacuum	1.998 (0.284)	0.68, 0.18	6.11	2.355 (0.001)	0.48, 0.39	0.38
	protein	2.082 (0.281)	0.66, 0.18	5.96	2.272 (0.006)	0.52, 0.34	0.86

Table S11. NEVPT2 energies (in eV) of the first and second excited states of PSII reaction center pigments in vacuum and in the protein electrostatic environment, using QM/MM optimized geometries for each pigment i.e. geometries **C** (QM | MD), as defined in the computational details. The calculations were performed with the SA-CASSCF(8,7) calculations with 9 roots included in the state-averaged orbital optimization. Oscillator strengths are indicated in parentheses. The state contributions of the leading configurations and the corresponding transition dipole moments (in Debye) are also given.

		S_1			S_2		
			Contributions of transitions:			Contributions of transitions:	
		E	H→L, H−1→L+1	$\|\mu\|$	E	H−1→L, H→L+1	$\|\mu\|$
P_{D1}	vacuum	1.988 (0.267)	0.64, 0.18	5.95	2.232 (0.016)	0.46, 0.33	1.38
	protein	1.990 (0.275)	0.62, 0.17	6.03	2.216 (0.042)	0.47, 0.31	2.25
P_{D2}	vacuum	1.966 (0.256)	0.67, 0.18	5.85	2.257 (0.003)	0.45, 0.36	0.63
	protein	1.981 (0.250)	0.63, 0.17	5.77	2.208 (0.050)	0.46, 0.33	2.44
Chl_{D1}	vacuum	1.990 (0.274)	0.65, 0.17	6.02	2.227 (0.018)	0.46, 0.33	1.47
	protein	1.943 (0.291)	0.64, 0.16	6.28	2.199 (0.048)	0.49, 0.30	2.39
Chl_{D2}	vacuum	1.981 (0.256)	0.64, 0.17	5.84	2.236 (0.019)	0.44, 0.35	1.50
	protein	1.968 (0.260)	0.64, 0.17	5.90	2.235 (0.030)	0.45, 0.34	1.87
$Pheo_{D1}$	vacuum	1.966 (0.204)	0.61, 0.20	5.22	2.423 (0.008)	0.46, 0.36	0.95
	protein	2.084 (0.135)	0.45, 0.20	4.13	2.488 (0.069)	0.43, 0.22	2.70
$Pheo_{D2}$	vacuum	1.979 (0.202)	0.61, 0.20	5.19	2.409 (0.010)	0.47, 0.35	1.03
	protein	2.143 (0.115)	0.51, 0.23	3.76	2.375 (0.072)	0.52, 0.25	2.83

Table S12. Lowest energy excited state (Q_y, in eV) of PSII reaction center pigments in the protein electrostatic environment and the corresponding electrochromic shifts calculated with different computational approaches. The calculations were performed using QM/MM optimized geometries for each pigment, i.e. geometries **C** (QM | MD), as defined in the computational details.

	S_1 in Protein (eV)				Electrochromic Shift (eV)			
	CASSCF (4,5)-7 / NEVPT2	CASSCF (8,7)-9 / NEVPT2	TD-DFT[a]	DLPNO-STEOM-CCSD[b]	CASSCF (4,5)-7 / NEVPT2	CASSCF (8,7)-9 / NEVPT2	TD-DFT[a]	DLPNO-STEOM-CCSD[b]
P_{D1}	1.968	1.990	1.959	1.613	-0.005	0.002	-0.010	-0.020
P_{D2}	1.959	1.981	1.946	1.620	-0.003	0.015	-0.009	-0.015
Chl_{D1}	1.928	1.943	1.913	1.575	-0.047	-0.047	-0.050	-0.067
Chl_{D2}	1.955	1.968	1.931	1.624	-0.011	-0.013	-0.021	-0.025
$Pheo_{D1}$	2.063	2.084	2.187	1.743	0.088	0.118	0.132	0.142
$Pheo_{D2}$	2.082	2.143	2.222	1.768	0.084	0.164	0.119	0.177

[a]TD-DFT calculations were performed with the ωB97X-D3(BJ) functional. [b]Values from Sirohiwal et al. *J. Phys. Chem. B* **2020**, *124*, 8761-8771.

Table S13. ωB97X-D3(BJ) TD-DFT energies (in eV) of the first excited state (S_1) of PSII reaction center pigments in vacuum and in the protein electrostatic environment, electrochromic shifts ($\Delta E = E_{protein} - E_{vacuum}$) and relative site energies referenced to Chl_{D1}. The calculations for each pigment were performed using geometries **A** (XRD | XRD), **B** (QM | XRD), and **C** (QM | MD), as defined in the computational details. QM geometry optimizations for the H atoms and for the QM regions in models **B** and **C** were performed with the PBE0 functional.

	**A** (XRD | XRD)				**B** (QM | XRD)				**C** (QM | MD)			
	$S_{1,vac.}$	$S_{1,prot.}$	Shift	E_{rel}	$S_{1,vac.}$	$S_{1,prot.}$	Shift	E_{rel}	$S_{1,vac.}$	$S_{1,prot.}$	Shift	E_{rel}
P_{D1}	1.870	1.843	-0.027	0.002	1.947	1.922	-0.025	-0.016	1.969	1.959	-0.010	0.046
P_{D2}	1.850	1.843	-0.007	0.002	1.976	1.981	0.005	0.043	1.955	1.946	-0.009	0.033
Chl_{D1}	1.886	1.841	-0.045	0.000	1.975	1.938	-0.037	0.000	1.963	1.913	-0.050	0.000
Chl_{D2}	1.878	1.843	-0.035	0.002	1.965	1.925	-0.040	-0.013	1.952	1.931	-0.021	0.018
$Pheo_{D1}$	2.125	2.200	0.075	0.359	2.022	2.148	0.126	0.21	2.055	2.187	0.132	0.274
$Pheo_{D2}$	2.128	2.218	0.090	0.377	2.063	2.195	0.132	0.257	2.103	2.222	0.119	0.309

Table S14. ωB97X-D3(BJ) TD-DFT energies (in eV) of the first excited state (S_1) of PSII reaction center pigments in vacuum and in the protein electrostatic environment, electrochromic shifts ($\Delta E = E_{protein} - E_{vacuum}$) and relative site energies referenced to Chl_{D1}. The calculations for each pigment were performed using geometries **A** (XRD | XRD), **B** (QM | XRD), and **C** (QM | MD), as defined in the computational details. QM geometry optimizations for the H atoms and for the QM regions in models **B** and **C** were performed with the PBE functional.

	**A** (XRD | XRD)				**B** (QM | XRD)				**C** (QM | MD)			
	$S_{1,vac.}$	$S_{1,prot.}$	Shift	E_{rel}	$S_{1,vac.}$	$S_{1,prot.}$	Shift	E_{rel}	$S_{1,vac.}$	$S_{1,prot.}$	Shift	E_{rel}
P_{D1}	1.870	1.843	-0.027	0.002	1.880	1.860	-0.020	0.004	1.897	1.887	-0.010	0.065
P_{D2}	1.850	1.842	-0.008	0.001	1.898	1.901	0.003	0.045	1.881	1.871	-0.010	0.049
Chl_{D1}	1.885	1.841	-0.044	0.000	1.898	1.856	-0.042	0.000	1.875	1.822	-0.053	0.000
Chl_{D2}	1.878	1.841	-0.037	0.000	1.896	1.857	-0.039	0.001	1.884	1.862	-0.022	0.040
$Pheo_{D1}$	2.123	2.199	0.076	0.358	1.881	2.016	0.135	0.160	1.899	2.043	0.144	0.221
$Pheo_{D2}$	2.126	2.216	0.090	0.375	1.910	2.049	0.139	0.193	1.938	2.074	0.136	0.252

Table S15. SA-CASSCF(4,5)-7/NEVPT2 energies (in eV) of the first excited state (S_1) of PSII reaction center pigments in vacuum and in the protein electrostatic environment, electrochromic shifts, and relative site energies referenced to Chl_{D1}. The calculations for each pigment were performed using geometries **A** (XRD | XRD), **B** (QM | XRD), and **C** (QM | MD), as defined in the computational details. QM geometry optimizations for the H atoms and for the QM regions in models **B** and **C** were performed with the PBE0 functional.

	**A** (XRD | XRD)				**B** (QM | XRD)				**C** (QM | MD)			
	$S_{1,vac.}$	$S_{1,prot.}$	Shift	E_{rel}	$S_{1,vac.}$	$S_{1,prot.}$	Shift	E_{rel}	$S_{1,vac.}$	$S_{1,prot.}$	Shift	E_{rel}
P_{D1}	1.886	1.856	-0.030	-0.010	1.956	1.942	-0.014	-0.008	1.973	1.968	-0.005	0.040
P_{D2}	1.869	1.860	-0.009	-0.006	1.978	1.979	0.001	0.029	1.962	1.959	-0.003	0.031
Chl_{D1}	1.911	1.866	-0.045	0.000	1.991	1.950	-0.041	0.000	1.975	1.928	-0.047	0.000
Chl_{D2}	1.900	1.869	-0.031	0.003	1.977	1.947	-0.030	-0.003	1.966	1.955	-0.011	0.027
$Pheo_{D1}$	2.022	2.062	0.040	0.196	1.960	2.033	0.073	0.083	1.975	2.063	0.088	0.135
$Pheo_{D2}$	1.977	2.055	0.078	0.189	1.977	2.068	0.091	0.118	1.998	2.082	0.084	0.154

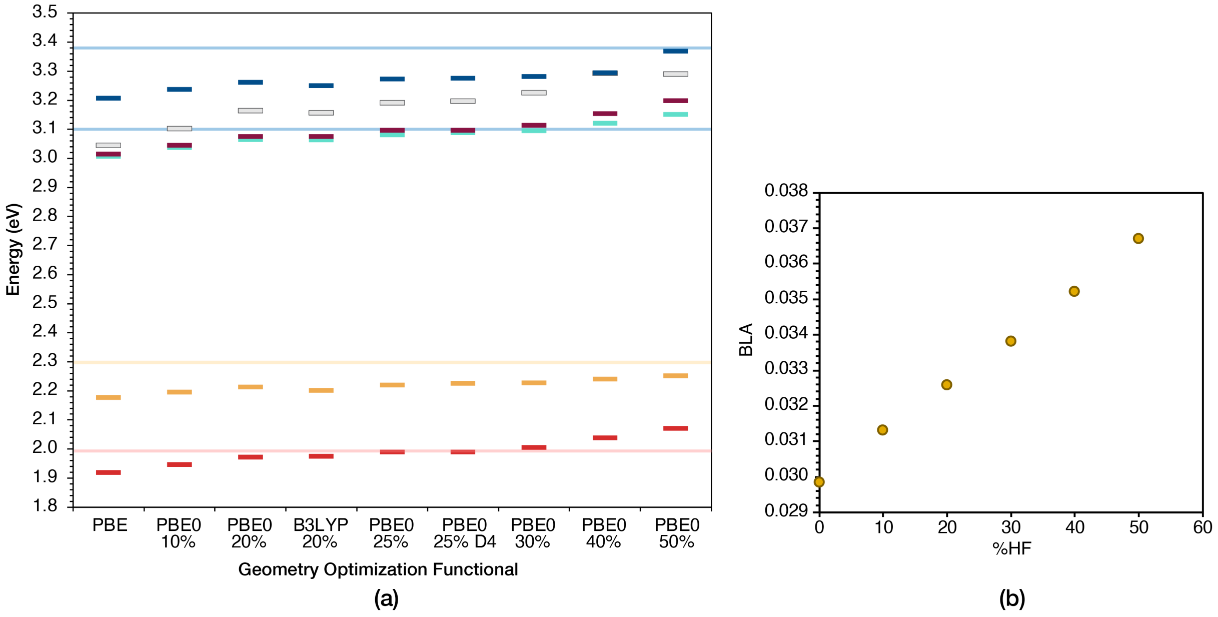

Figure S9. a) SA-CASSCF(4,5)-7/NEVPT2 excitation energies of Chl *a* gas-phase optimized using the PBE0 functional with different % Hartree-Fock (HF) exchange, from 0% to 50%, B3LYP, and PBE0 with D4 dispersion corrections. Optimization with B3LYP (default, 20% HF) and PBE0 with 20% gives the same excitation energies, which suggests that HF exchange is the factor that affects the systematic shift of the excitation energies. Moreover, optimization with PBE0 (default, 25% HF) without and with D4 dispersion corrections also give the same results. b) Correlation between the bond length alternation (BLA) in the optimized structures of Chl *a* with the %HF exchange used in the optimization functional. BLA is defined as the difference between mean bond lengths of formal double bonds and single bonds.
